# A *Trypanosoma cruzi* trans-sialidase peptide demonstrates high serological prevalence among infected populations across endemic regions

**DOI:** 10.1172/JCI199277

**Published:** 2026-02-05

**Authors:** Hannah M. Kortbawi, Ryan J. Marczak, Jayant V. Rajan, Nash L. Bulaong, John E. Pak, Wesley Wu, Grace Wang, Anthea Mitchell, Aditi Saxena, Aditi Maheshwari, Rachel Alfaro Leone, Charles J. Fleischmann, Emily A. Kelly, Evan Teal, Rebecca L. Townsend, Susan L. Stramer, Emi E. Okamoto, Jacqueline E. Sherbuk, Eva H. Clark, Robert H. Gilman, Rony Pedro Colanzi, Efstathios D. Gennatas, Caryn Bern, Joseph L. DeRisi, Jeffrey D. Whitman

**Affiliations:** 1 Department of Biochemistry and Biophysics,; 2 Medical Scientist Training Program,; 3 Department of Epidemiology and Biostatistics, and; 4 Department of Laboratory Medicine, UCSF, San Francisco, California, USA.; 5 Chan Zuckerberg Biohub San Francisco, San Francisco, California, USA.; 6 Scientific Affairs, American Red Cross, Gaithersburg, Maryland, USA.; 7 New York University School of Medicine, New York, New York, USA.; 8 Section of Infectious Diseases, Department of Medicine and; 9 Division of Tropical Medicine, Department of Pediatrics, Baylor College of Medicine, Houston, Texas, USA.; 10 Johns Hopkins Bloomberg School of Public Health, Baltimore, Maryland, USA.; 11 Universidad Catolica Boliviana, Santa Cruz, Bolivia.; 12 Department of Medicine, UCSF, San Francisco, California, USA.

**Keywords:** Immunology, Infectious disease, Antigen, Diagnostics, Parasitology

## Abstract

**BACKGROUND:**

Infection by *Trypanosoma cruzi*, the agent of Chagas disease, is endemic to the Americas and can irreparably damage the cardiac and gastrointestinal systems during decades of parasite persistence. Diagnosis of chronic infection requires confirmation by multiple serological assays due to the imperfect performance of existing tests. Current serology tests were developed using small specimen sets predominantly from South America, and lower performance has been observed in patients who acquired infection in Central America and Mexico.

**METHODS:**

To improve Chagas disease serology, we evaluated antibody responses against the entire *T*. *cruzi* proteome with phage display immunoprecipitation sequencing and further evaluated high-prevalence antigens by immunoassay. We utilized specimen sets representing Mexico, Central America, and South America and varying cardiac disease presentations, from 185 cases and 143 controls.

**RESULTS:**

We identified over 1,300 antigenic *T*. *cruzi* peptides. A trans-sialidase antigen demonstrated high seroprevalence across all regions and has not previously been described as a diagnostic target to our knowledge. Orthogonal validation of this peptide demonstrated increased antibody reactivity for infections originating from Central America.

**CONCLUSION:**

This study provides proteome-wide identification of seroreactive *T*. *cruzi* peptides across a range of endemic populations not previously represented in antigen discovery and identifies a trans-sialidase peptide antigen (TS23) with potential for translation into diagnostic serological assays.

**FUNDING:**

Chan Zuckerberg Biohub, the Chan Zuckerberg Biohub Physician-Scientist Fellowship Program, the NIH National Heart Lung and Blood Institute award K38HL154203, and the NIH Eunice Kennedy Shriver National Institute of Child Health and Human Development award F30HD117526.

## Abstract 2

Serology tests for Chagas disease demonstrate different performance by geographic region. This study evaluates antibody responses against *Trypanosoma cruzi* to improve diagnostics across infected populations.

## Introduction

Chagas disease is caused by infection with the protozoan parasite, *Trypanosoma cruzi*, which is transmitted by triatomine insect vectors. The disease is endemic to the Americas, with vector-borne transmission occurring in suitable ecological zones of Latin America (1). In the United States, the major disease burden occurs among Latin American immigrant populations exposed in their birth countries, though rare autochthonous infections have been documented in Texas, California, Arizona, Tennessee, Mississippi, and other southern states (2, 3). Chronic Chagas disease is considered a lifelong infection without treatment. *T*. *cruzi* can infect many nucleated cell types but causes pathology in the cardiac and gastrointestinal systems. An estimated 20% to 30% of people with chronic Chagas disease develop symptoms of end organ damage after years to decades of infection. Related cardiac presentations include cardiac conduction system deficits, dilated cardiomyopathy, and sudden cardiac death (4). Ten percent of infected individuals may develop gastrointestinal dysmotility disorders (5, 6). Because this parasite is predominantly intracellular in the chronic phase and symptoms are largely nonexistent or nonspecific, detection of anti–*T*. *cruzi* antibodies in peripheral blood is the most sensitive method for diagnosis and is the only reliable means of screening asymptomatic patients.

The test performance of current Chagas disease serology assays does not have sufficient accuracy (sensitivity or specificity) to effectively diagnose patients by one test alone, and clinical diagnosis based on symptoms is not possible because most infections are asymptomatic or nonspecific (7). Pan American Health Organization/World Health Organization guidelines require confirmation by 2 tests with distinct antigen sources. The indications for *T*. *cruzi* serology span many areas of health care, including clinical diagnosis, blood donor screening, and solid organ or hematopoietic stem cell transplant donor and recipient testing (8–11). In practice, securing additional confirmatory testing for patients and identifying clinical laboratories that offer more than one serology test can be difficult and time-consuming, and ultimately patients may be lost to follow-up. Given the mounting awareness of the need for Chagas disease screening and imperfect test performance, it is clear that the serology assays themselves must be improved to increase the effectiveness of screening and diagnosis efforts.

Recent studies evaluating regionally diverse Chagas disease populations highlight differential reactivity to commercial *T*. *cruzi* serology assays between infected populations, with the lowest reactivity in individuals from Mexico, intermediate reactivity from Central America, and the highest reactivity from South America (12–15). Up to an estimated 10% loss in sensitivity between infections originating from Mexico compared with South America has been observed depending on the assay (12). Other studies based in endemic areas have documented decreased performance of commercial serology tests in regions of Mexico and Central America, as well as Peru (16–19). *T*. *cruzi* is a genetically diverse parasite, currently classified into 6 genetic lineages or discrete typing units (DTUs: TcI–TcVI) (20), plus a potential seventh, bat-associated genotype (TcBat), most closely related to TcI (21). It is hypothesized that host immunological responses and antigenic differences between regional *T*. *cruzi* strains may be the basis of the differential serological responses. However, the areas with problematically low reactivity to commercial assays are largely found where TcI is predominant, but not all TcI-predominant areas show low reactivity (22). Genetic variation is also high within TcI (23), suggesting that DTU-level classification is not sufficiently granular to map host immunology to parasite genetics.

The antigens used in current commercial diagnostics originated from a surge of Chagas disease serology research over the last 3 decades (24). These studies tended to rely on screening with sera from high-prevalence regions of South America, where TcII/V/VI are predominant, mainly Brazil and Argentina. Since then, more robust techniques for antigen discovery have emerged in the form of high-density peptide microarrays. Recent application of these techniques to Chagas disease have generated additional antigen targets (25–27). However, these studies used pooled sera for determining the initial downselection of antigens for secondary peptide array libraries. Such an approach is unable to discern commonality of antigens across the entire *T*. *cruzi* proteome among the pooled sera. The antigen targets chosen for follow-on validation in individual specimens were therefore biased toward the highest reactivity antigens within a pool, not necessarily the highest prevalence antigens.

To address these gaps, we employed phage display immunoprecipitation sequencing (PhIP-seq) (28) using a synthetic oligonucleotide library with high-density coverage of the *T*. *cruzi* proteome using 47 amino acid (aa) peptides. We performed immunoprecipitation using 185 serology-confirmed cases from geographically diverse regions of Latin America to explicitly represent the genetic diversity of *T*. *cruzi* antigens across DTUs, as well as varying presentations of Chagas cardiomyopathy to control for any differences by disease severity. The goal of this study was to employ a next-generation antigen discovery technique to *T*. *cruzi* and evaluate high-prevalence antigen targets with translational potential for serological diagnostics.

## Results

### Development of T. cruzi proteome library.

**Figure 1:**
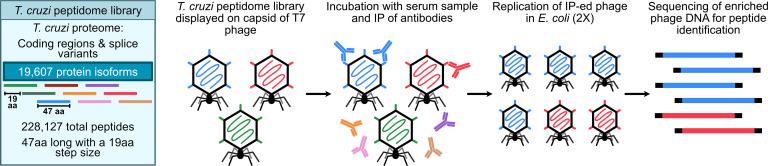
PhIP-seq library design and assay steps. Phage library displays the proteome of *T*. *cruzi* in 47 aa peptides with a 19 aa step size on the capsid of T7 phage. The library includes all coding regions of the proteome and splice variants. We performed the PhIP-seq assay by incubating the phage library with human plasma, followed by immunoprecipitation of antibodies in the sample and enrichment of antibody-bound phage through lysis in *E*. *coli*. We performed 2 rounds of enrichment and then sequenced the enriched phage to obtain the identity of the immunoprecipitated peptides.

We constructed a T7 phage display library to display the entire *T*. *cruzi* CL Brener proteome in 49 aa peptides with a 19-mer overlap in consecutive sequences (Methods, Figure 1). The library includes 228,127 *T*. *cruzi* peptides that represent 19,607 proteins. Over 99.6% of the ordered peptides were represented in the final cloned library, with 90% of peptides represented within a 9-fold difference of read counts (Supplemental Figure 1; supplemental material available online with this article; https://doi.org/10.1172/JCI199277DS1).

### PhIP-seq identifies antibodies against T. cruzi peptides.

We performed PhIP-seq on peripheral blood samples from 3 distinct specimen sets including US blood donors with routine Chagas disease serology screening (BD; *n* = 90; *n* = 64 seropositive, *n* = 26 seronegative), a Chagas disease cardiac biomarker study (CBM; *n* = 143; *n* = 121 seropositive, *n* = 22 seronegative), and independent healthy controls (New York Blood Center [NYBC]; *n* = 95). See Methods for a full description of these specimens. The PhIP-seq library contained human glial fibrillary acidic protein (GFAP) sequences, so a polyclonal anti-GFAP antibody was used as a positive control for immunoprecipitation. Positive control samples were highly enriched for GFAP peptides (Supplemental Figure 2). We excluded 10 samples from the analysis because of low sequencing read counts: 7 seropositive CBM samples, 2 seronegative CBM study samples, and 1 NYBC control. In total, 185 cases and 143 controls were included for further data analysis.

We used a conservative analysis approach to identify antibody reactivity to individual *T*. *cruzi* peptides that were enriched (*z*-score ≥ 5) in at least 5% of seropositive patients (Supplemental Figure 3). *Z*-scores were calculated based on the distribution of sequencing reads per 100,000 (RPK) value for a given peptide in seronegative patients, which included endemic-region seronegative controls as well as independent seronegative controls from the United States. With this approach, 5,638 individual peptides representing 4,001 proteins were enriched in the seropositive CBM samples, and 8,710 peptides representing 5,629 proteins were enriched in the seropositive BD samples. Between both cohorts, 12,978 antigenic peptides corresponding to 7,373 unique proteins were identified, with an overlap of 1,370 peptides across 961 proteins in both Chagas disease study sets (Supplemental Figure 4). A total of 67 and 85 peptides were reactive in at least 70% of samples among the BD samples and CBM samples, respectively. Across both cohorts, 43 of these 70% seroreactivity peptides were shared.

### Enriched T. cruzi antigens in seropositive samples.

**Figure 2:**
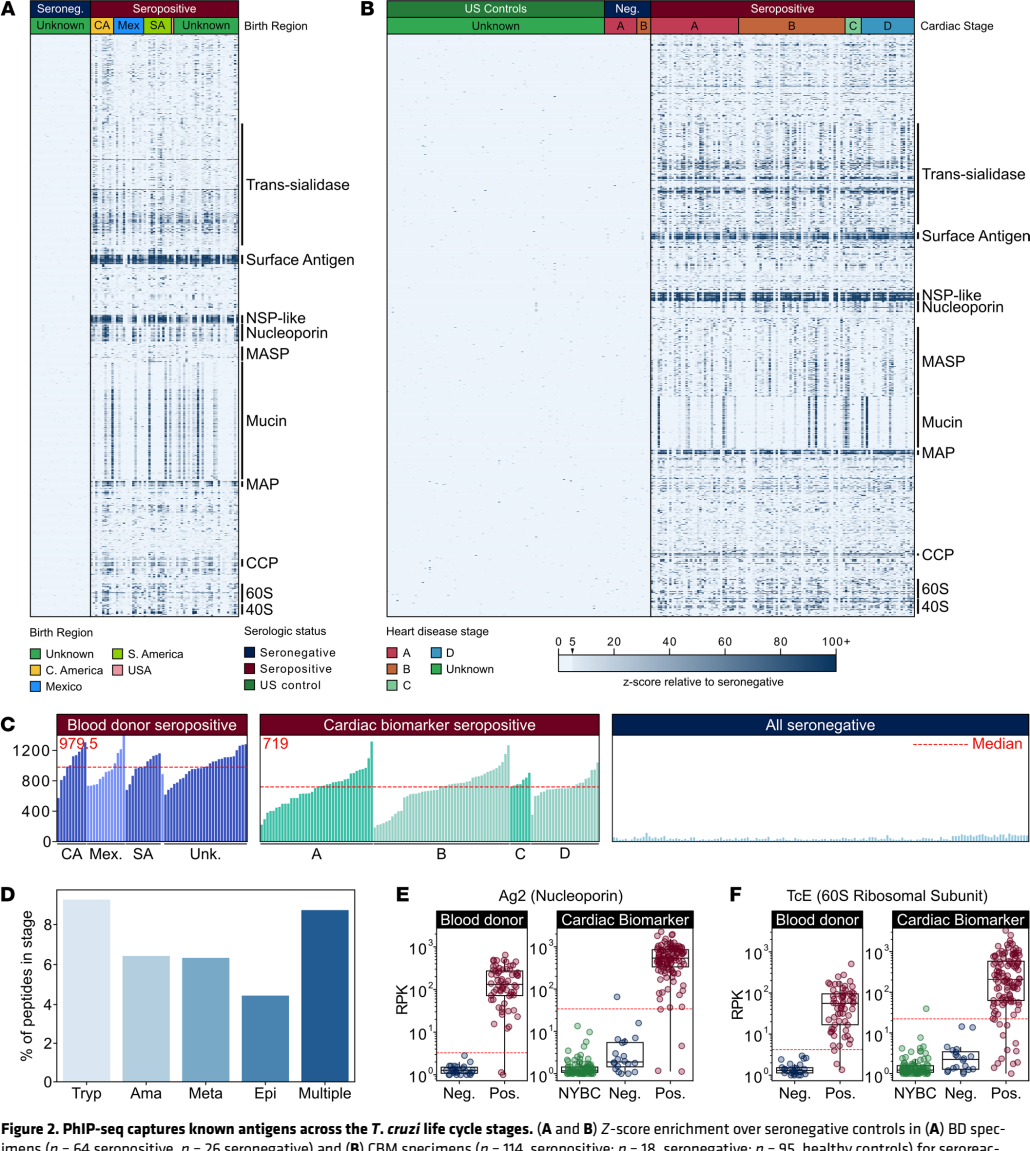
PhIP-seq captures known antigens across the *T*. *cruzi* life cycle stages. (**A** and **B**) *Z*-score enrichment over seronegative controls in (**A**) BD specimens (*n* = 64 seropositive, *n* = 26 seronegative) and (**B**) CBM specimens (*n* = 114, seropositive; *n* = 18, seronegative; *n* = 95, healthy controls) for seroreactive peptides (rows) with >15% seropositivity within each cohort sorted by protein name (peptides) and patient region of origin (samples) (*n* = 10, Central America; *n* = 13, Mexico; *n* = 12, South America; *n* = 1, United States; *n* = 28, unknown) (**A**) or cardiac disease stage (*n* = 14, stage A seronegative; *n* = 6, stage B seronegative; *n* = 38, stage A seropositive; *n* = 46, stage B seropositive; *n* = 7, stage C seropositive; *n* = 23, stage D seropositive) (**B**). Protein groups with well-characterized antigens: surface antigen, surface antigen 2 [CA-2]; NSP-like, nucleoporin NSP1-like C-terminal domain-containing protein; MASP, mucin-associated surface protein; mucin, TcMUCII; MAP, microtubule-associated protein; CCP, calpain-like cysteine peptidase; 60S, 40S, ribosomal subunit proteins. (**C**) Breadth of antibody reactivity, shown as seroreactive peptides in each person. Samples are grouped by geographic region or heart disease stage. (**D**) Peptides identified as seroreactive that are part of proteins expressed in stages of the *T*. *cruzi* life cycle (Tryp, trypomastigote; Ama, amastigote; Meta, metacyclic trypomastigote; Epi, epimastigote; Multiple, trypomastigote, amastigote, and/or metacyclic trypomastigote stages). Stage expression analysis shows seroreactive peptides in every host-interfacing life cycle stage. (**E** and **F**) Selected known seroreactive antigens are captured by *T*. *cruzi* PhIP-seq. NYBC is US controls. Antibody reactivity to 2 known antigens, (**E**) Ag2, a nucleoporin protein, and (**F**) TcE, a 60S ribosomal subunit protein (RPK). Dotted red line signifies the RPK that corresponds to a *z*-score cutoff of 5 in the seronegative population of each cohort.

The antigenic peptides identified across both specimen sets represented 38% of the 19,607-member proteome of *T*. *cruzi*. Most of these peptides demonstrated no enrichment in seronegative samples (Figure 2, A and B). The median number of enriched Chagas disease–specific peptides in each seropositive sample was higher in the BD specimens compared with the CBM specimens (Figure 2C), but the mean number of enriched peptides per sample did not significantly differ within cohorts by patient region of origin (BD specimens) or by heart disease stage (CBM specimens) [Kruskal-Wallis, BD *H*(2) = 1.77, *P* = 0.41; CBM *H*(3) = 2.49, *P* = 0.48].

The proteins from which the enriched peptides derive are predominantly expressed in the host phase life cycle stages of *T*. *cruzi* (metacyclic trypomastigotes, trypomastigotes, and amastigotes) (29) (Figure 2D). There were relatively fewer seroreactive peptides that corresponded to proteins expressed in the epimastigote form, which occurs only in the gut of the triatomine vector but is used as the source of parasite lysate antigens in commercial assays. Examples of host phase–specific proteins that had high antibody reactivity included trans-sialidases and mucin-associated surface proteins.

### Identification of high-prevalence T. cruzi antigens across Latin America.

To identify high-prevalence antigens shared across endemic regions representing different *T*. *cruzi* DTUs, we first analyzed BD specimens, which included individuals born in Mexico, Central America, and South America. We performed 2 complementary approaches to identify individual antigens with sufficient seroprevalence to have utility for clinical diagnostic assays. First, we used the *z*-scored, peptide-level data to identify peptides enriched in at least 90% of seropositive BD samples. Second, we used mass univariate analysis to create a ranked list of top antigenic peptides by largest predictor coefficient values. The mass univariate analysis approach models peptide RPK scores based on diagnostic status in our BD specimens. All peptides identified by the *z*-score approach were also identified as significantly enriched by the mass univariate analysis (Supplemental Figure 5). These analyses were then performed on the CBM specimens to evaluate for any differences in high-prevalence antigens by cardiac disease status. None were identified.

**Figure 3:**
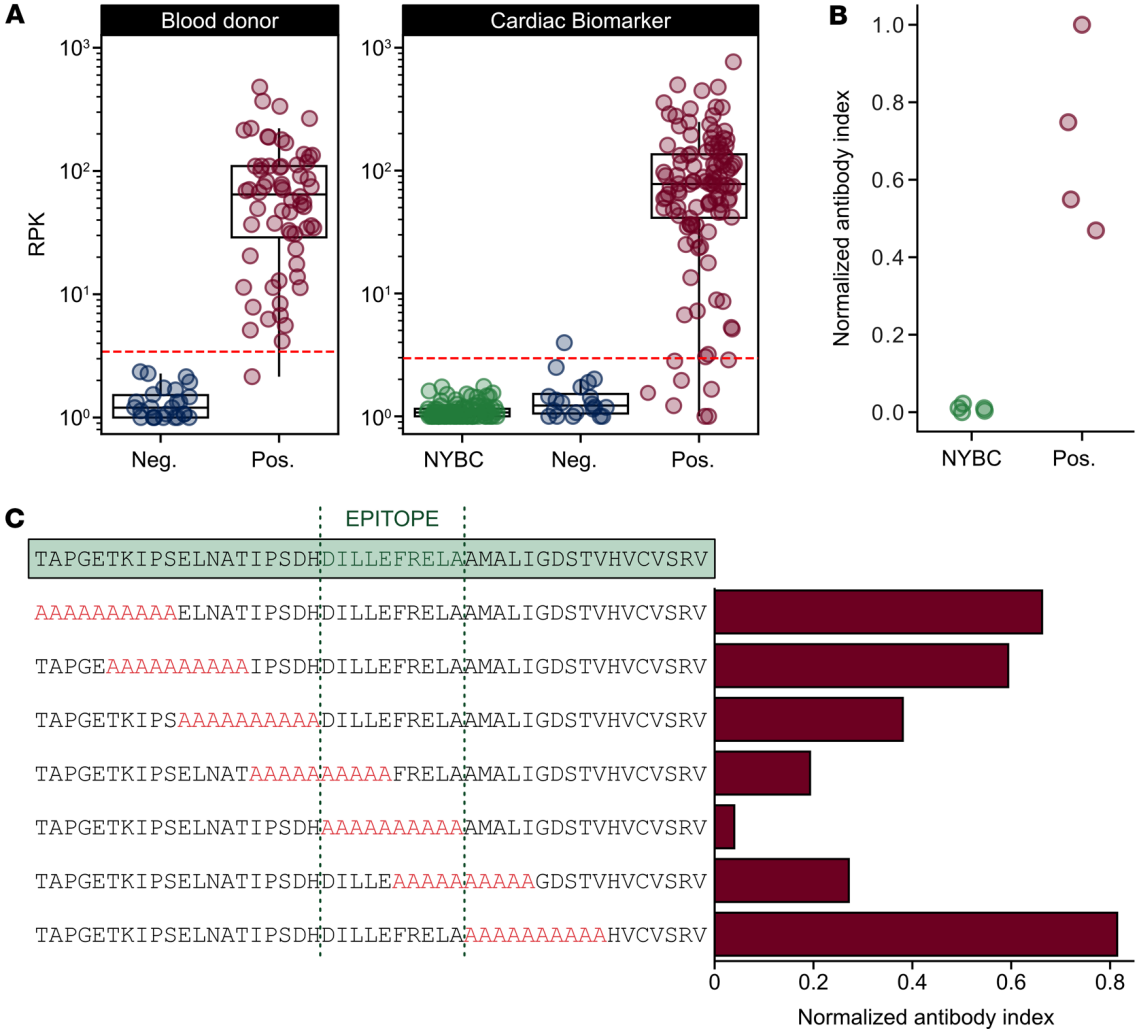
A trans-sialidase peptide sequence is a highly reactive serological antigen. (**A**) Anti–trans-sialidase peptide antibody reactivity is plotted as RPK from BD and CBM specimen sets. The dotted red line signifies the RPK that corresponds to a *z*-score cutoff of 5 in the seronegative population of each cohort. (**B**) Trans-sialidase reactivity orthogonal validation using a split-luciferase binding assay (SLBA). Reactivity was tested against 4 seropositive BD specimens and 5 seronegative US healthy control specimens. (**C**) Alanine-scanning mutagenesis in 10 aa windows (highlighted in red) across the entire trans-sialidase antigenic fragment demonstrates the seroreactive epitope in Chagas disease. Values are normalized antibody indices and represent the averages of 5 seropositive BD specimens.

These analyses yielded 23 peptides (Supplemental Table 1), including 20 peptides that all contain the repetitive PFGQAAAGDKPS antigenic sequence, present in a current diagnostic antigen known as Ag2 (30, 31) (Figure 2E). An additional highly reactive peptide, which contained the antigenic sequence KAAAPKKAAAPQ, has high sequence homology to another known diagnostic antigen, TcE (32) (Figure 2F). The final 2 high-prevalence peptides belonged to the trans-sialidase family (Figure 3A) and shared the sequence APGETK[V/I]PSELNATIPSDHDILLEFR[D/E]LAAMALIG. To our knowledge, this peptide has not previously been used in a serological diagnostic.

### Epitope mapping and validation of the high-prevalence trans-sialidase antigen.

To orthogonally validate antibody reactivity to the high-prevalence trans-sialidase peptide, we performed a split-luciferase binding assay (SLBA). Briefly, we generated the trans-sialidase peptide with a C-terminal HiBiT tag and immunoprecipitated it with plasma from 4 seropositive BD patient samples with the highest PhIP-seq RPK values and 5 independent healthy US controls. Incubation of the immunoprecipitated peptides with LgBiT produces luminescence as a quantitative measurement of antibody binding. Four Chagas disease–seropositive samples with high PhIP-seq enrichment were reactive to the trans-sialidase peptide, while seronegative control samples were not (Figure 3B).

A sequential alanine-scan was performed to further map the reactive epitope of the trans-sialidase peptide. Using samples from 5 seropositive BD individuals, we determined that the critical region for immunoreactivity was a 10 aa sequence that spans positions 648 to 658 of the full-length trans-sialidase protein (DILLEFRELA) (Figure 3C). To be permissive of the epitope, we chose a final antigenic sequence of IPSDHDILLEFRELA, corresponding to the 2 alanine blocks with the lowest reactivity, henceforth referred to as TS23. Basic local alignment search tool (BLAST) analysis of TS23 in National Center of Biotechnology Information (NCBI) database identified 37 proteins from all *T*. *cruzi* entries with more than 93% (15 aa) sequence identity, all of which were from trans-sialidase or putative trans-sialidase genes.

### Evaluation of trans-sialidase antigen TS23 antibody reactivity.

**Figure 4:**
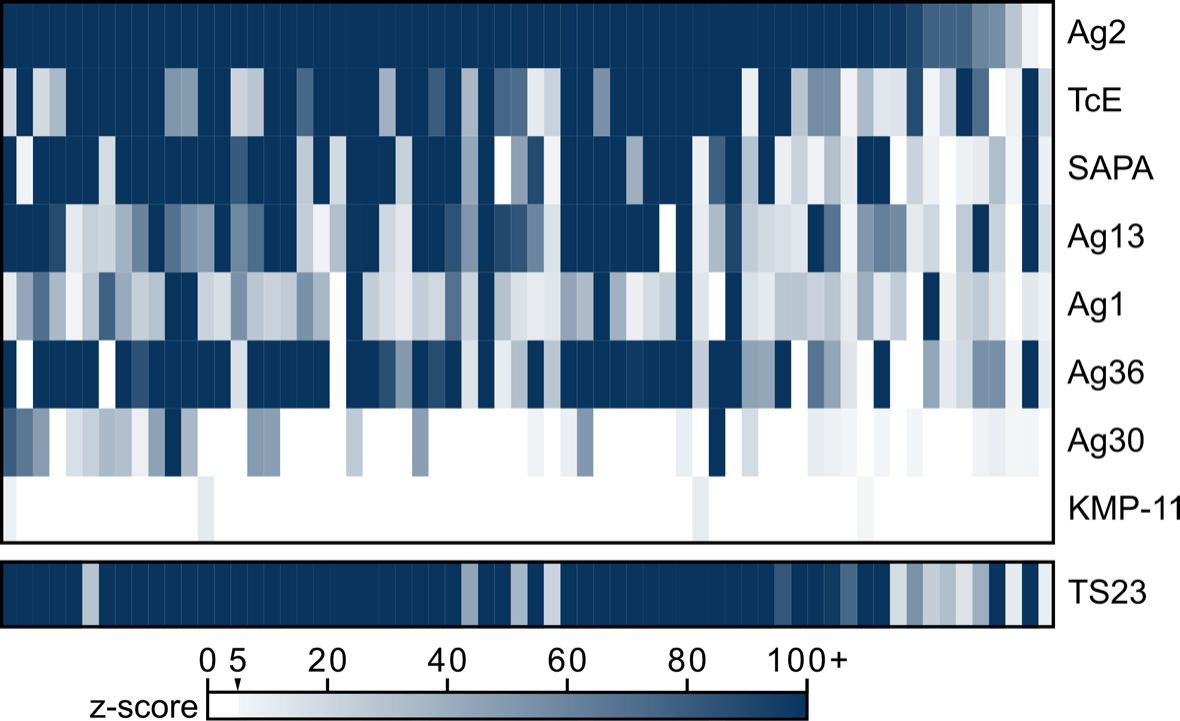
PhIP-seq antibody reactivity of current diagnostic antigens and TS23 in Chagas disease–seropositive specimens. Heatmap of *z*-score enrichment over seronegative controls in the seropositive BD specimens (*n* = 64) of recombinant antigens in current FDA-cleared serology tests. Recombinant antigens in current tests include Ag1, Ag2, Ag13, Ag30, Ag36 (33, 34), shed acute phase antigen (SAPA) (35), KMP-11 (36), TcD, and TcE (30, 37). Note that TcD contains the same antigenic epitope as Ag13. Each antigen motif was derived using MEME and then scored against the entire *T*. *cruzi* PhIP-seq proteome. The maximum *z*-score across all peptides with significant sequence matches to a given antigen motif was plotted for each sample and each antigen.

To evaluate the potential of TS23 as a diagnostic serology antigen, we compared its PhIP-seq performance with that of current diagnostic antigens. To address the fact that current diagnostic antigen sequences vary at certain amino acids from available sequencing data (33) and the specific antigen sequences used in commercial diagnostics are not publicly available for all assays, we agnostically derived the amino acid sequence motifs of 8 diagnostic antigens used in US Food and Drug Administration–cleared (FDA-cleared) serology tests (24) using Multiple EM for Motif Elicitation (MEME) (see Methods) (Supplemental Figure 6) (34–36). These motifs were then queried against the entire *T*. *cruzi* PhIP-seq proteome using FIMO to identify all peptides with a significantly similar sequence to each antigen (37). The maximum *z*-score for each BD sample across all PhIP-seq peptides with a sequence match to a given antigen motif was plotted (Figure 4). The maximum *z*-score across all peptides with a sequence match to TS23 was also shown to compare this antigen’s reactivity with those in used in current diagnostics. Any sample with a *z*-score of at least 5 for a peptide that contained an antigen motif was considered enriched for antibody reactivity prevalence calculations (Supplemental Table 2). TS23 had high prevalence, demonstrated in 100% (64/64) of the seropositive BD samples and 95% (108/114) of CBM seropositive samples. By comparison, only Ag2 and TcE had similar antibody reactivity and prevalence across seropositive specimens. In contrast, other antigens in commercial diagnostics, Ag1, Ag13, and Ag36, had similar prevalence but lower reactivity, and KMP-11 was rarely enriched.

**Figure 5:**
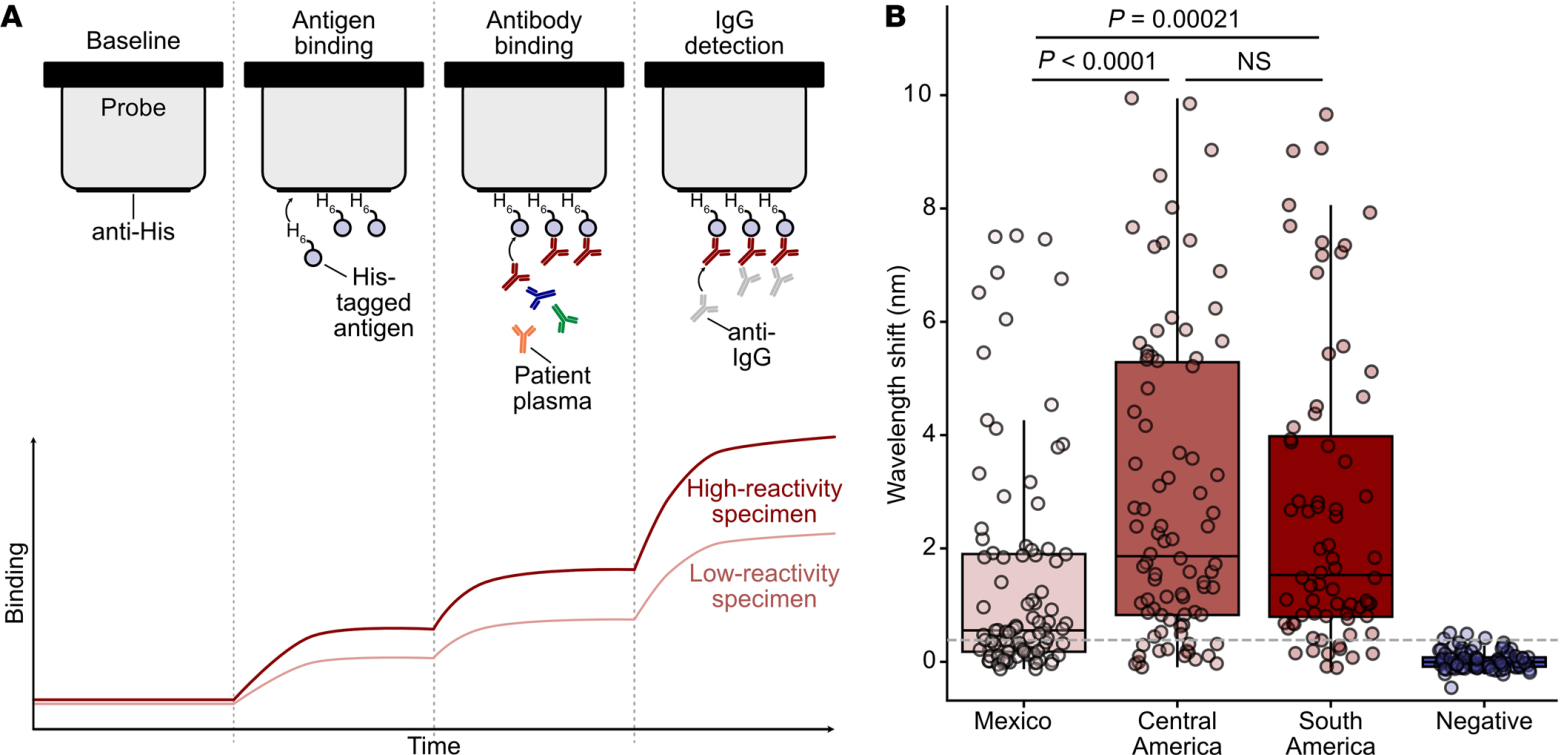
Biolayer interferometry validates trans-sialidase antigen TS23 seroreactivity in a large cohort. (**A**) Schematic of biolayer interferometry (BLI) approach. BLI uses a fiber-optic probe to measure the wavelength of light reflected from the surface of a biosensor, which changes because of light interference when an analyte binds. First, an anti-His tag probe is incubated with His-tagged antigen. Then, the probe is incubated in diluted serum or plasma, and antibodies bind the immobilized antigen on the probe. To quantify IgG-specific reactivity, anti-IgG antibodies are added and bind the immobilized patient antibodies. (**B**) Seropositive BD specimens (*n* = 250) demonstrate a range of reactivity to the trans-sialidase antigen by quantitative BLI immunoassay, while seronegative BD specimens (*n* = 86) do not (Wilcoxon’s rank-sum test). Reactivity, as denoted by wavelength shift, was higher in Central American (*n* = 86) and South American (*n* = 72) specimens than in Mexican specimens (*n* = 92). Dashed line corresponds to the 25th percentile across all seropositive specimens. Associations between BLI reactivity were tested using Kruskal-Wallis tests; significant results were followed with a post hoc MWU test with a correction for multiple comparisons using the Bonferroni method.

To further validate and directly characterize the antibody reactivity of TS23, we performed a quantitative IgG biolayer interferometry (BLI) immunoassay on 335 BD specimens with previous Chagas disease serology testing (*n* = 250, seropositive; *n* = 85, seronegative) (Methods, Figure 5A). Seropositive samples were chosen to include all specimens with region-of-origin data (Mexico, *n* = 92; Central America, *n* = 86; South America, *n* = 72) (Figure 5B). A Kruskal-Wallis test showed that the difference in reactivity was significantly different between regions [*H*(3) = 154.76, *P* < 0.0001]. The results of post hoc pairwise comparisons between regions demonstrated seroreactivity (BLI nanometer [nm] shift) was lower in individuals from Mexico compared with Central America (Mann-Whitney *U* [MWU]; *P* < 0.0001) and South America (MWU; *P* = 0.00021). Specimens from individuals from Central America and South America did not show differences in reactivity (MWU; *P* = 1.000); however, the median BLI nm shift was higher for individuals from Central America (1.86) compared with South America (1.53). Seronegative specimens did not demonstrate any overt nonspecific reactivity to the TS23 antigen.

To evaluate if specimens with low reactivity to TS23 are weakly reactive overall, we compared TS23 reactivity with previous results from an FDA-cleared Chagas disease serology ELISA (Chagatest Recombinante v.3.0, Wiener Labs [Wv3]). This assay contains a multiepitope recombinant antigen composed of Ag1, Ag2, Ag13, Ag30, Ag36, and SAPA (30, 31). Analysis of the 25th percentile of TS23 BLI reactivity yielded 63 specimens originally positive by blood donor testing. The reactivity (signal-to-cutoff ratio) of the Wv3 ELISA was in the 25th percentile of previous testing (12) for 70% (44/63) of the low-reactivity TS23 BLI specimens. This comparison with previous Wv3 ELISA test results suggests that most low-reactivity TS23 specimens are weakly reactive specimens overall. Breakdown of these specimens by region of origin included 60% (38/63) from Mexico, 24% (15/63) from Central America, and 16% (10/63) from South America.

### Performance of a multiepitope antigen including TS23.

**Figure 6:**
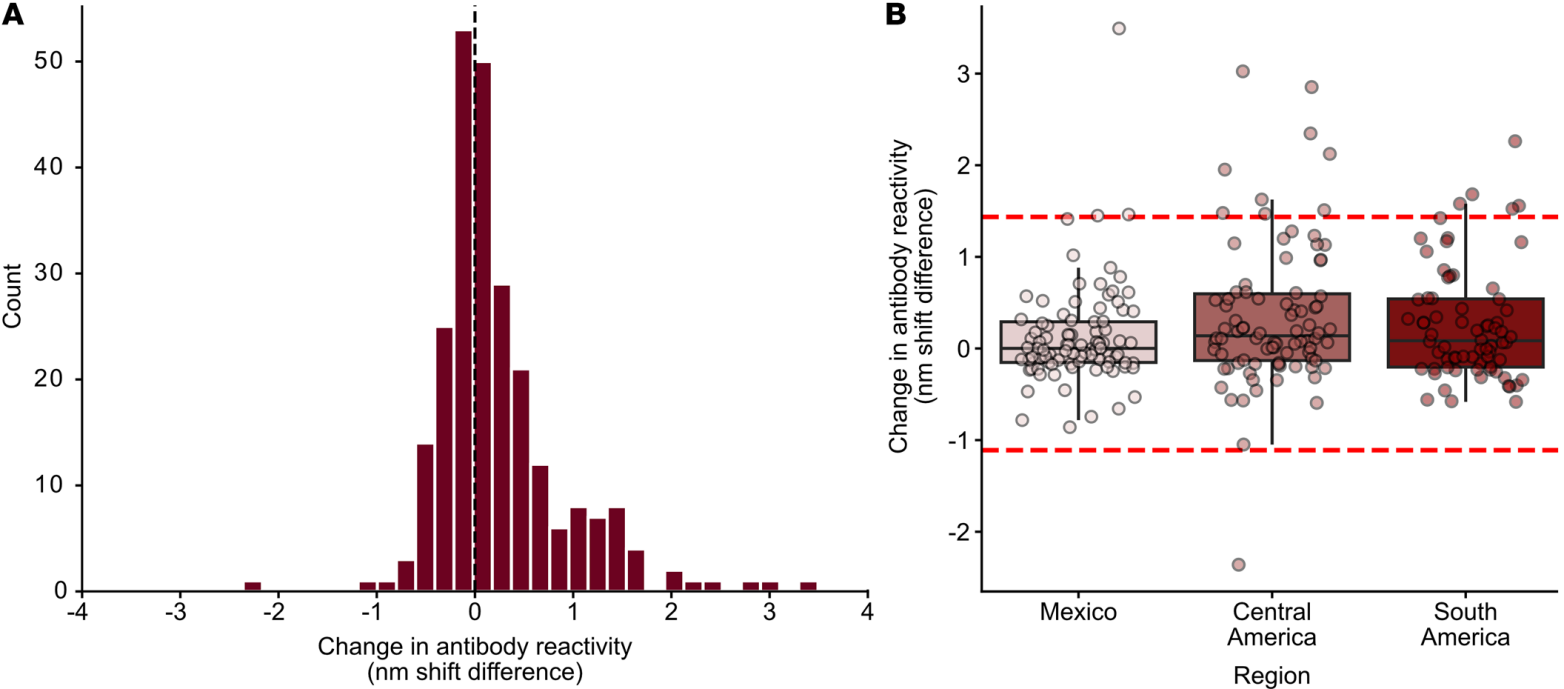
Distribution of difference in antibody reactivity between a multiepitope *T*. *cruzi* antigen with and without TS23 using BLI. (**A**) Histogram of reactivity difference of TcMulti+TS23 – TcMulti demonstrates a positive skew of 1.4 gained from the addition of the TS23 antigen (D’Agostino-Pearson test for normality, *P* < 0.0001, K2 84.29). (**B**) The change in TcMulti reactivity with the addition of TS23 shows the reactivity gains from TcMulti+TS23 are predominantly in patients with infections originating from Central America, though the change is not significantly different between regions [Kruskal-Wallis, *H*(2) = 4.67, *P* = 0.097]. Dashed red lines represent 1.5× IQR ± quartile cutoff.

To assess whether TS23 increases test performance, we developed a multiepitope antigen based on the MEME analysis of classical *T*. *cruzi* antigens used in current diagnostics (Supplemental Figure 6), with and without TS23, referred to as TcMulti+TS23 and TcMulti, respectively. TcMulti contains 1 copy of each peptide antigen, except for 2 repeats of the short sequences of TcE and Ag13, to allow for direct stoichiometric comparisons of reactivity. The performance of TcMulti and TcMulti+TS23 were compared head-to-head by BLI immunoassay using the same set of specimens for TS23 alone to evaluate whether the inclusion of the TS23 antigen in the multiantigen construct leads to reactivity increases that exceed the natural variability observed among positive samples. Among the positive specimens, the difference in BLI immunoassay reactivity (nm shift) between TcMulti+TS23 and TcMulti had a positive skew of 1.4 (D’Agostino-Pearson test for normality, *P* < 0.0001, K2 84.29) (Figure 6A). The median difference of reactivity between TcMulti+TS23 and TcMulti for seropositive samples was 0.07 (IQR, –0.16, 0.49). We used 1.5× IQR above and below the quartiles as the cutoff to identify outliers, of which 15 (6%) seropositive samples were greater than Q3 + 1.5× IQR, while only 1 sample (0.04%) was below Q1 – 1.5× IQR; a Kruskal-Wallis test showed that the difference in reactivity between multiepitopes (TcMulti+TS23 – TcMulti) did not differ significantly between regions [*H*(2) = 4.67, *P* = 0.097] (Figure 6B). The percentage increases in signal gained by TcMulti+TS23 compared with TcMulti for the positive outliers ranged from 115% to 648.7%.

**Figure 7:**
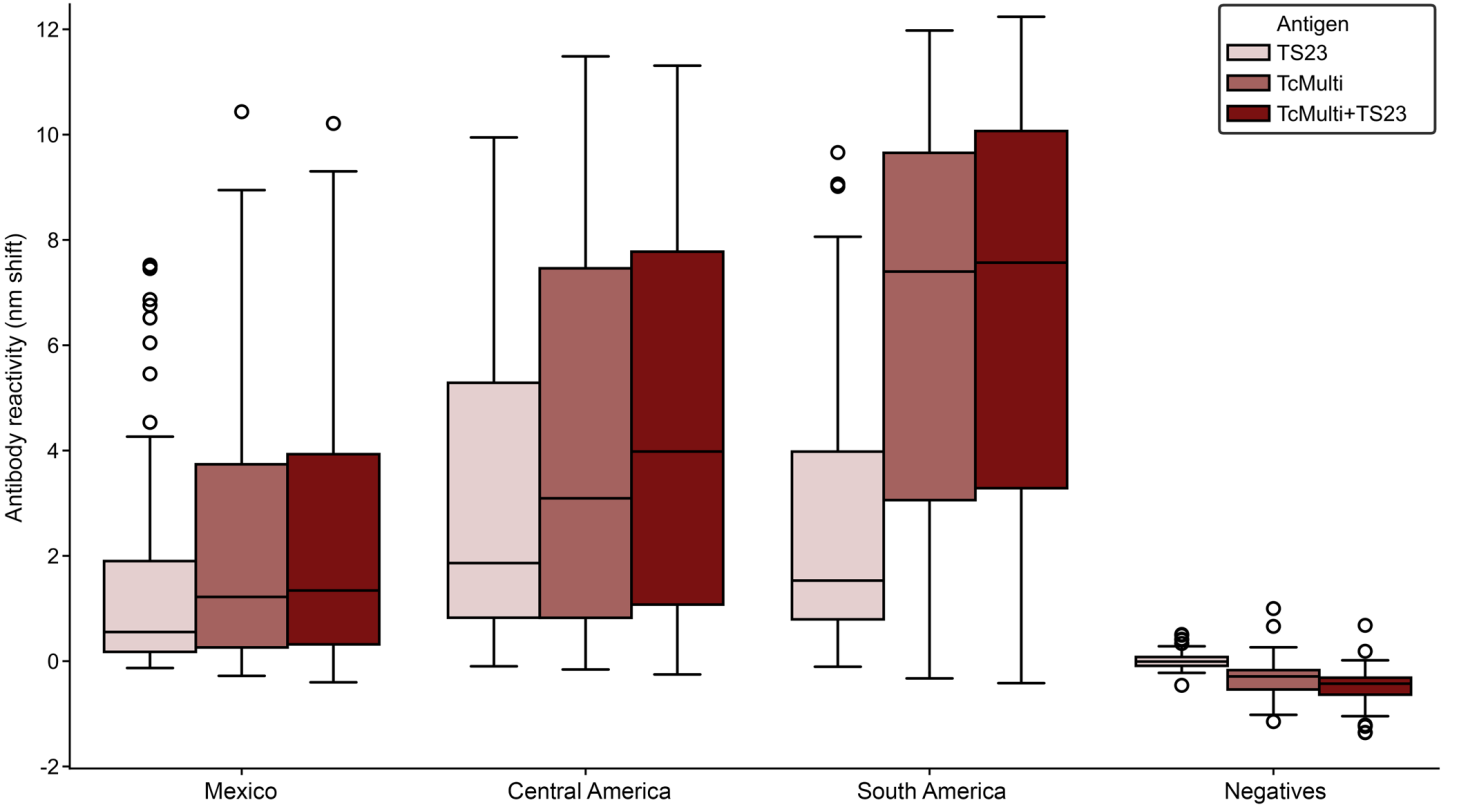
BLI using TS23 and multiepitope antigens demonstrates differential seroreactivity by region of infection. Box plot of seroreactivity (nm shift), measured by BLI, to *T*. *cruzi* antigens using seropositive (*n* = 250) and seronegative (*n* = 85) BD specimens. TS23 demonstrates the highest reactivity in infections from Central America (*n* = 86), while the multiepitope constructs of classical antigens (TcMulti) with and without TS23 demonstrate the highest reactivity in infections from South American infections (*n* = 72). Infections from Mexico (*n* = 92) have lower reactivity to all antigens.

When comparing antibody reactivity between multiepitope constructs and TS23 alone among endemic regions, different patterns of distributions emerged (Supplemental Tables 3 and 4). TS23 demonstrates the highest relative reactivity in specimens originating from Central America, while the multiepitope constructs (with and without TS23) demonstrated the highest reactivity in South American specimens (Figure 7), which corresponds to previous observations in commercial assays containing similar antigens (12, 13, 15).

To evaluate the diagnostic test performance of TS23, TcMulti+TS23 versus TcMulti, we performed receiver operating characteristic curve analysis of BLI reactivity with a stratified 70/30 train/test split (Supplemental Figure 7). Both multiepitope antigens demonstrated excellent discrimination, with training set 5-fold cross-validated average AUCs of 0.983 (TcMulti+TS23) and 0.982 (TcMulti), and test set AUCs of 0.986 and 0.984, respectively. TS23 had a training set average of 0.949 and a test set AUC of 0.869.

A fixed-cutoff approach to determine qualitative test performance (sensitivity and specificity to original blood donor testing result) was calculated using 2 standard deviations above the average reactivity of seronegative specimens to TcMulti+TS23 and TcMulti as the threshold for positive seroreactivity, yielding a nm shift cutoff 0.27. Despite the quantitative analysis above showing antibody reactivity increases from TcMulti+TS23, the qualitative test performance was equivalent between multiepitope constructs with and without TS23, demonstrating approximately 85% sensitivity and 98% specificity (Supplemental Table 5). However, it should be noted that this assay has not been optimized for diagnostic testing.

## Discussion

Chagas disease is a neglected tropical disease endemic to the Americas, affecting over 6 million people worldwide (1). Numerous diagnostic challenges for chronic Chagas disease exist, including low clinical awareness, nonspecific or asymptomatic presentation, and imperfect test performance (38, 39). In addition to better testing for populations with epidemiological risk factors for exposure to triatomine vectors, effective testing is needed for populations from endemic areas presenting for prenatal screening, blood donation, and organ transplant evaluation (8–11). The major disparities for Chagas disease serology testing are insufficient sensitivity and specificity of any one diagnostic test alone to confirm infection and differential serological reactivity by region of origin of *T*. *cruzi* infection.

To address this, we carried out our *T*. *cruzi* PhIP-seq study in 2 well-characterized Chagas disease specimen sets, which represent the largest number of samples evaluated for *T*. *cruzi* antigen discovery to our knowledge. One set (BD) includes specimens collected via blood donation in the United States but with known country of origin to infer the infection’s region of origin between Mexico, Central America, and South America (12). An additional set (CBM) is derived from a Bolivian study with varying stages of Chagas cardiomyopathy, collected via a clinical study of cardiac biomarkers (40). Analysis of these 2 specimen sets by PhIP-seq identified more than 1,300 reactive *T*. *cruzi* peptides highly specific to individuals with Chagas disease. Downselection on common antigens among Chagas disease–seropositive individuals filtered to only 3 antigenic sequences with sufficient prevalence for diagnostic utility (enrichment in at least 90% of specimens). This vast difference between total reactive peptides and high-prevalence antigens illustrates the uniqueness of the adaptive immune system response within an individual host. By contrast, the rare high-prevalence antigens warrant further research into the host-pathogen biology that drives their immunodominance.

Two of these high-prevalence antigens were identified by multiple groups from early phage display studies and are already incorporated into current serological diagnostics: Ag2 (30, 31), a nucleoporin protein, and TcE (41), a 60S ribosomal subunit protein. The third and, in fact, most prevalent peptide antigen discovered in our study, TS23, has not previously been described as a serological diagnostic antigen and belongs to the trans-sialidase family. Trans-sialidase enzymes comprise a unique pathogenic and antigenic family of secreted and glycophosphatidylinositol-anchored cell surface proteins in *T*. *cruzi*. Their primary functional activity is to transfer sialic acids from mammalian cells to β-galactosidase residues on *T*. *cruzi’s* cell membrane to assist in cell invasion (42). Trans-sialidases also appear to modulate the host immune response, likely acting as decoy antigens (43). The evidence for this relates to the discovery of the SAPA, a multirepeat antigen located at the C-terminal end of trans-sialidase enzymes (44). Since the catalytic end is in the N-terminal region, it is hypothesized that C-terminal antigens evolved to protect the trans-sialidase enzyme activity from humoral responses. BLAST analysis of the trans-sialidase antigen identified by our study showed 37 *T*. *cruzi* proteins with more than 93% sequence identity, all of which were trans-sialidase or putative trans-sialidase genes. All sequences were located at the C-terminal end, matching with the immunodominant nature of known antigens from this family. The identification of TS23 as a highly prevalent trans-sialidase peptide antigen introduces questions about what relative proportion of antibody reactivity signal TS23 contributes compared with other trans-sialidase antigens, such as the SAPA and trypomastigote excreted-secreted antigens (TESA). It will be very informative to investigate the concordance of TS23, SAPA, and TESA reactivity in future studies related to diagnostic test performance.

Our findings also demonstrate that the TS23 antigen identified in this study has increased antibody reactivity in specimens from individuals born in Central America, compared with previous analyses of the same specimens by current clinical diagnostics (12). Beyond the potential implications for endemic populations in Central America, identification of TS23 is important for screening and diagnosis in the United States, considering that a large proportion of the Latin American immigrant population is predominantly from El Salvador and Guatemala (2). While this is a promising advancement, unfortunately, TS23 does not have improved seroreactivity in individuals who acquired *T*. *cruzi* infection in Mexico. Further study is needed to evaluate whether this is due to lower anti–*T*. *cruzi* IgG levels in individuals exposed in Mexico or the absence of regional *T*. *cruzi* antigens of Mexican origin. Posttranslational protein glycosylation may be a source of unique antigenicity outside of the primary peptide sequence (45).

An interesting observation can be made from the relative comparison of antibody reactivity distributions between multiepitope constructs and TS23 alone. The TcMulti antigen described in this study was derived empirically from available sequencing data of known *T*. *cruzi* antigens used in current diagnostics. Previous studies evaluating commercial assays based on these antigens have demonstrated different antibody reactivity by region in infection, with the lowest in Mexico, intermediate in Central America, and highest in South America (12, 13, 15). It has been hypothesized that the original phage display studies that discovered these classical antigens may be biased toward South American infections, considering the specimens and *T*. *cruzi* strains used in this discovery work were primarily from South America (24). Our current study demonstrates that this pattern holds true for our classical multiepitope antigen backbone (TcMulti), while TS23, which was discovered in our specimen set that contains a large proportion of individuals born in Central America, has the highest reactivity from this region. This finding strengthens the hypothesis that test performance is affected by antigenic diversity between regional *T*. *cruzi* genotypes and that sampling bias toward a specific region in discovery work may affect test performance, depending on the population. It is imperative that future antigen discovery work include as many endemic populations as possible toward a goal of a universal antigen (or combination of antigens) that will generate optimal test performance regardless of the region of infection. Future screening of TS23 reactivity across larger and more diverse infected populations will further elucidate the contributions of TS23 reactivity to improve diagnostic detection in groups with low reactivity to existing antigens.

Last, while our final antigenic construct, TcMulti+TS23, has good diagnostic potential, it does not outperform current commercial assays with an approximate sensitivity and specificity of 85% and 98%, respectively (Supplemental Table 5). However, the immunoassay developed in this study was designed to measure quantitative differences in antibody reactivity with a higher dynamic range than ELISA for regional comparisons, not as a diagnostic test. Follow-on studies across multiple immunoassay formats are planned to optimize serum dilution and secondary antibody concentration to improve test accuracy. Evaluations of antigen cross-reactivity with other related infections or inflammatory diseases and interfering substances, such as free hemoglobin (hemolysis) and high triglycerides (hyperlipidemia), are also necessary to validate an assay to clinical standards.

Our study is limited by retrospective selection bias on samples tested by current diagnostic assays. Prospective testing in at-risk populations will be important to further validate the TS23 antigen and will allow for a more objective comparison of the diagnostic sensitivity of TS23 compared with other antigens. Ultimately, to create the ideal serologic test for diagnosis of chronic Chagas disease, we would combine the fewest recombinant targets for a multiepitope antigen that approaches 100% sensitivity and specificity to eliminate the need for confirmatory testing for all initial *T*. *cruzi*–seropositive results. Such a test would greatly facilitate Chagas disease screening, diagnosis, and treatment.

In summary, our study has discovered a *T*. *cruzi* trans-sialidase peptide antigen (TS23) not used in current diagnostics that is serologically reactive and prevalent across endemic countries of Latin America and varying degrees of cardiac disease severity. It demonstrates the highest reactivity among infections acquired from Central America. Future studies will prioritize immunoassay optimization for diagnostic uses and evaluate the real-world test performance in multiple immunoassay formats common in clinical laboratories, with the goal of clinical translation.

## Methods

### Sex as a biological variable.

Our study examined male and female humans, and similar findings are reported for both sexes.

### Study design.

Samples used in this study included blood donor plasma collected within the United States and serum from clinical research collected in Bolivia. The blood donor plasma samples were provided in collaboration with the American Red Cross (ARC) with sample selection criteria described previously (12). All BD specimens were confirmed by blood donor testing assays and algorithms (9). A subset of specimens was used for antigen discovery experiments (*n* = 90; *n* = 64, seropositive; *n* = 26, seronegative). Region-of-origin data were available for 35 specimens (*n* = 13, Mexico; *n* = 10, Central America; *n* = 12, South America). The serum samples from clinical research studies in Bolivia were collected as part of a CBM study (40). Specimens were collected from a large public hospital in Santa Cruz, tested and confirmed for *T*. *cruzi* serostatus, and further stratified for cardiac status by clinical assessment, electrocardiogram (ECG), and echocardiography (echo) studies. In total, 143 serum samples were included for this study, including 22 seronegative (*n* = 15, without cardiac abnormalities [Stage A]; *n* = 7, with cardiac abnormalities [Stage B]) and 121 seropositive (*n* = 40, without cardiac abnormalities [Stage A]; *n* = 81, with cardiac abnormalities [Stages B–D]). Cardiac staging is defined as A, normal ECG, normal echo; B, abnormal ECG, normal echo; C, 40%–55% ejection fraction (EF) by echo, normal left ventricular end diastolic diameter (LVEDD); and D, EF < 40% or LVEDD > 57 mm. Specimens were randomized to 96-well plates prior to testing and frozen at –20°C. A separate set of *T*. *cruzi*–seronegative plasma specimens (*n* = 95) from the NYBC was used as an independent negative control for antigen discovery experiments with the CBM specimen set.

### Construction of T. cruzi phage library.

Reference protein sequences for the *T*. *cruzi* strain CL Brener assembly GCF_000209065.1 (46) were obtained from the NCBI site. All sequences in the peptidome were processed using a previously described bioinformatic pipeline (47). Briefly, all full-length protein sequences were decomposed into a series of overlapping peptides. Each peptide was 47 aa in length with consecutive peptides overlapping by 19 aa. The full set of peptides was collapsed using the command line tool cd-hit (48, 49) at 90% sequence similarity, resulting in a final set of 228,127 peptides spanning the *T*. *cruzi* peptidome (19,607 proteins). Peptides tiling over the length of the GFAP were added to the library as a positive control for immunoprecipitation. Peptide sequences were converted to their coding DNA sequences with common 5′ (GTAGCTGGTGTTGTAGCTGCC) and 3′ (GGTGACTACAAGGATGATGATGATAAA) linker sequences appended to each peptide encoding sequence. The 3′ linker sequence encoded a FLAG tag. The final library, consisting of 228,162 peptides that correspond to 19,608 proteins, was ordered from Agilent Technologies.

### Cloning and packaging into T7 phage.

The oligo pool was received in a single tube, lyophilized, and resuspended to 0.2 nM. The pool was amplified using Phusion polymerase (New England Biolabs [NEB]) and linker-specific primers (TAGTTAAGCGGAATTCAGTAGCTGGTGTTGTAGCTGCC, ATCCTGAGCTAAGCTTTTTATCATCATCATCCTTGTAGTCACC). The amplified library was purified using Ampure XP magnetic beads (Beckman Coulter) and confirmed to have a single-size product by gel electrophoresis. A total of 1 μg of the cleaned library was then digested using EcoRI-HF and HindIII-HF restriction enzymes (NEB) and purified again using Ampure XP beads. Digestion of the library product was confirmed by visualizing a 20 bp size shift using the Bioanalyzer High Sensitivity DNA Analysis kit (Agilent). The digested library was cloned into T7 Select vector arms (Novagen 70550-3) as previously described (47). Four packaging reactions were performed and then pooled. The final phage library was propagated in BLT5403 *E*. *coli* (Novagen 70550-3).

### Immunoprecipitation of antibody-bound phage.

PhIP-seq was performed using the *T*. *cruzi* peptide phage display library with plasma or serum samples using our previously published PhIP-seq protocol (https://www.protocols.io/view/derisi-lab-phage-immunoprecipitation-sequencing-ph-4r3l229qxl1y/v1). Patient plasma was diluted 1:1 in storage buffer (0.04% NaN_3_, 40% glycerol, 40 mM HEPES [pH 7.3], 1× PBS [–Ca and –Mg]) to preserve antibody integrity. A total of 1 μL of diluted plasma was incubated with 500 μL of the input phage display library for the first round of immunoprecipitation. Positive control immunoprecipitations were performed using 1 μL of 1:10 diluted anti-GFAP antibody (Dako, Z0334) (Supplemental Figure 2). We used 10 μL of Dynabeads Protein A/G slurry (Thermo Fisher Scientific) per sample. After 1 round of immunoprecipitation, phages were amplified in *E*. *coli* and enriched in a second round of immunoprecipitation. The final lysate was spun and stored at 4°C for next-generation sequencing library prep. Immunoprecipitated phage lysate was heated to 70°C for 15 minutes to expose DNA. DNA was then prepared for next-generation sequencing in 2 subsequent PCR amplifications. The final prepared libraries were sequenced using an Illumina sequencer to a read depth of approximately 1 million reads per sample.

### PhIP-seq data analysis.

Sequencing reads from FASTQ files were aligned to the reference *T*. *cruzi* peptide library, and individual peptide counts were normalized to RPK by dividing by the sum of counts and multiplying by 100,000 to account for varying read depth. All subsequent analyses were performed using Python (version 3.12.2) unless otherwise noted.

To identify Chagas disease–specific enriched peptides and avoid false positives, a conservative analysis pipeline was used as follows. Peptide-level enrichment across known seronegative samples was calculated and used to generate *z*-scores ([x-mean seronegative]/standard deviation seronegative) for the Chagas disease–seropositive and –seronegative and NYBC control samples. The *z*-score for any seronegative sample was calculated by leaving out that sample from the mean of seronegative samples for each peptide. A moving threshold analysis was implemented to determine the *z*-score threshold and the number of patients with Chagas disease that must share enrichment to a given peptide to completely differentiate seropositive and seronegative patients (Supplemental Figure 3). Based on this analysis, *z*-score cutoff of 5 and shared enrichment across at least 5% of Chagas disease samples (*n* ≥ 3 BD specimens; *n* ≥ 5 CBM specimens) and 1 or fewer seronegative samples was set for hit calling.

Additional validation of the *z*-score approach was executed using a mass univariate analysis using generalized linear models applied to each peptide. Peptide fragments with uniform values across all samples were removed due to lack of variability. RPK values were scaled by subtracting the mean and dividing by the standard deviation calculated within each peptide. Scaled RPK values for each peptide were regressed on Chagas disease diagnostic status (*y_i_* = *b_0i_* *+* *b_1i_* *×* *x*) where *y_i_* is the scaled RPK value, *b_0i_* is the intercept of the *i*-th peptide fragment, *b_1i_* is the predictor coefficient, and *x* is the diagnostic status in BD samples or cardiac disease stage in CBM samples. The resulting coefficient quantified the strength and direction of the association between diagnostic status (or disease stage) and the scaled RPK values for each peptide, where positive coefficient values represent, on average, a higher RPK for that peptide in seropositive specimens. Analyses were performed using R (version 4.3.1).

Antigenic prevalence of a *T*. *cruzi* peptide was calculated as the number of seropositive samples enriched for a specific peptide divided by the number of seropositive samples in the respective specimen set (BD and CBM). High-prevalence antigens were designated as enrichment in ≥90% of seropositive specimens and no seronegative specimens.

Life cycle stage–specific analysis of seroreactive *T*. *cruzi* antigens is based on the Life cycle proteome (Brazil) data set from TriTrypDB. Gene IDs for stage-specific proteins were mapped onto the gene IDs that corresponded to seroreactive peptides.

### SLBA.

A high-prevalence antigen by PhIP-seq that was not already included in commercial diagnostics was selected for orthogonal validation by SLBA. A detailed SLBA protocol can be found online at https://www.protocols.io/view/split-luciferase-binding-assay-slba-protocol-4r3l27b9pg1y/v1 Briefly, the high-prevalence peptide antigen was inserted into a split luciferase construct containing a T7 promoter and a terminal HiBiT tag and synthesized as DNA oligomers (Twist Biosciences). The oligos were amplified using 5′-AAGCAGAGCTCGTTTAGTGAACCGTCAGA-3′ and 5′-GGCCGGCCGTTTAAACGCTGATCTT-3′ primer pair and purified using the DNA Clean and Concentrator-5 kit (Zymo). Purified PCR products were transcribed and translated in vitro (IVTT) using wheat germ extract (Promega L4140), and the Nano-Glo HiBiT Lytic Detection System (Promega, N3040) was used to quantify translated protein using relative luciferase units (RLU) detected on a luminometer. Background luminescence was calculated using an IVTT reaction that used a construct encoding a STOP codon 5′ of the HiBiT tag. Peptides were normalized to 2 × 10^7^ RLU per well, incubated overnight with patient plasma or a positive control mouse anti-HiBiT antibody (Promega, N7200), and immunoprecipitated with a Dynabeads Protein A/G bead slurry. The immunoprecipitation was washed 4 times with SLBA buffer (0.15 M NaCl, 0.02 M Tris-HCl pH 7.4, 1% w/v sodium azide, 1% w/v bovine serum albumin, and 0.15% v/v Tween 20), and remaining luminescence was measured using the Nano-Glo HiBiT Lytic Detection System in a luminometer. Antibody index was calculated as (RLU sample – RLU mock IP)/(RLU sample – RLU anti-HiBiT) for orthogonal validation of the trans-sialidase peptides. For epitope mapping by alanine-scanning mutagenesis, the antibody index was calculated as (RLU seropositive – RLU US control)/(RLU seropositive – RLU anti-HiBiT) and normalized to the antibody index of immunoprecipitation using the WT peptide sequence.

### MEME and FIMO motif analysis.

To empirically re-derive a selected diagnostic antigen motif, all BD-enriched peptides were filtered to those peptides that mapped to the antigenic protein (e.g., any enriched peptide that belonged to a nucleoporin protein for Ag2) These peptide sequences were queried using *MEME* (MEME 5.5.7) (https://meme-suite.org/meme/index.html) with the following *meme* command options and parameters: -protein -mod zoops -nmotifs 10 -minw 6 -maxw 15 -objfun classic -markov_order 0.

The derived motifs were then manually inspected to identify the motif that clearly matched the published diagnostic antigen sequences (Supplemental Figure 6) (24). This motif (or multiple motifs, if the antigen sequence was over 47 aa, as in the case of Ag1 and Ag36) was then queried against the entire *T*. *cruzi* PhIP-seq proteome using the following *fimo* command options and parameters: --thresh 1e-4 --qv-thresh.

The only exceptions to this analysis were antigens Ag13, TcE, and KMP-11. Ag13 and TcE are short, highly repetitive antigens and so were identified using the *meme* parameter -mod anr. The final antigenic motif identified for TcE was very short (6 aa) and thus required different *fimo* significance thresholds to identify similar sequences. A *q* value threshold of 1 × 10^–2^ was set for this antigen only. Finally, KMP-11 was represented by only 3 overlapping peptides that map to kinetoplastid membrane protein KMP-11 (XP_808865.1), so motif discovery was not possible. To look for sequence similarity across the *T*. *cruzi* proteome, the 92 aa KMP-11 protein was queried against the proteome using *blastp* (BLAST 2.12.0), and no other peptides with significant sequence similarity were identified. The 3 KMP-11 peptides alone were used for downstream analysis of KMP-11 antigen reactivity.

To assess the reactivity of patient samples against these antigen motifs, the maximum *z*-score across all peptides with a sequence match to a given antigen motif was plotted for each BD sample.

### Peptide antigen expression.

We selected a minimal antigenic peptide sequence that consisted of the 15 aa that, when mutated via alanine scanning, produced the lowest binding signal on SLBA (Figure 3C), to test using BLI. This peptide sequence was repeated 7 times in series to create a final protein that was approximately 13 kDa. The insert sequence was synthesized by Twist Bioscience in a pET-21(+) vector, with a C-terminal 6X-His tag and under control of a T7 promoter and lac repressor.

The expression plasmid was transformed into BL21(DE3) competent *E*. *coli* (Thermo Fisher Scientific) and plated onto Luria-Bertani (LB) agar plates containing carbenicillin. Isolates were expanded in 1 liter of LB broth with carbenicillin grown at 37°C to an OD_600_ of 0.6. The culture was induced with 1 mM isopropyl β-d-1-thiogalactopyranoside and grown at 25°C with shaking for another 18 hours. The cells were then centrifuged at 16,770*g* for 30 minutes at 4°C to collect the cell pellet.

A stock lysis buffer (20 mM sodium phosphate, 20 mM imidazole, 500 mM NaCl, 0.5 mM TCEP, 5% glycerol, pH 7.4) was made with EDTA-free protease inhibitor cocktail (cOmplete Protease Inhibitor, Roche) per 50 mL. The pelleted cells were resuspended in 100 mL of cold lysis buffer and run through an LM10 microfluidizer at 15,000 PSI for 5 cycles. The flow-through lysate was collected after each cycle and combined. The lysate was centrifuged at 24,105*g* for 30 minutes at 4°C. The supernatant was collected and filtered through a 0.22 μm vacuum filtration device.

Recombinant His-tagged antigen was purified from the filtered lysate using a Ni-NTA resin gravity flow column. After loading the lysate to the column, the column was washed with a wash buffer (20 mM sodium phosphate, 40 mM imidazole, 500 mM NaCl, 0.5 mM TCEP, pH 7.4). The antigen was eluted with an elution buffer (20 mM sodium phosphate, 500 mM imidazole, 500 mM NaCl, 0.5 mM TCEP, pH 7.4). Peptide yield from the purification was quantified using NanoDrop (Thermo Fisher Scientific), and the purity of the product was verified by protein gel electrophoresis. Expression of the peptide was confirmed by anti-His tag Western blot using a 6X-His tag monoclonal antibody (Invitrogen, MA1-21315).

### Multiepitope peptide expression.

The multiepitope peptide was designed using the antigenic motifs from Ag2, TcE, SAPA, Ag13, Ag1, AG36, and AG30 identified by MEME analysis (Supplemental Figure 6). The multiepitope without TS23 consisted of the following sequence: DKPSPFGQAAAGDKPKKAAKPKAAAKPSAHSTPSTPADSSAHAEPKPAEPKSSMNARAQELAREKKLADRAFLDQKPEGVPLRELPLDDDSDFVAMEQERRQQLEKDPRRNAKREIAALEEDVGPRHVDPDHFRTTQDAYRPVDPSAYKRKAAEATKVAEAEKQHHHHHH. The multiepitope with TS23 consisted of the following sequence: DKPSPFGQAAAGDKPKKAAKPKAAAKPSAHSTPSTPADSSAHAEPKPAEPKSSMNARAQELAREKKLADRAFLDQKPEGVPLRELPLDDDSDFVAMEQERRQQLEKDPRRNAKREIAALEEDVGPRHVDPDHFRTTQDAYRPVDPSAYKRKAAEATKVAEAEKQIPSDHDILLEFRELAHHHHHH.

The peptides were synthesized in the pET-21(+) vector and expressed and purified as described above.

### BLI serological immunoassay.

A GatorPrime analyzer (Gator Bio) was used to perform BLI to evaluate the antibody reactivity to the recombinant peptide antigen. BLI uses a fiber-optic probe to measure the wavelength of light (nm) reflected from the surface of a biosensor, which shifts in response to analyte binding (Figure 5A). Quantitative BLI serological immunoassay can be performed by measuring nm shift to antigen-bound probe incubated in diluted serum or plasma and subsequently in anti-human IgG for quantifying class-specific responses. BLI methodology was chosen for these analyses because it has a higher dynamic range for assessing antibody-antigen reactivity compared with traditional colorimetric ELISA (50). An anti–*T*. *cruzi* IgG BLI method was developed using a commercial *T*. *cruzi* Chimeric Chagas Multi-Antigen (MACH; Jena Biosciences). This is a polypeptide chain of 87 aa with epitopes from previously known antigens: Peptide 2, TcD, TcE, and SAPA, fused with a 6X-His tag. This BLI method was optimized using high-, intermediate-, and low-reactivity seropositive BD specimens previously determined by Chagatest Recombinante v.3.0 anti–*T*. *cruzi* ELISA (Wiener Labs), which contain the MACH antigens.

The anti–*T*. *cruzi* IgG BLI assay was adapted for the recombinant antigen discovered by PhIP-seq and for the multiepitope assay by varying the protein concentration to achieve saturation of nm shift signal of the anti-His tag fiber-optic probe (Supplemental Figure 8). The final method consisted of the following BLI conditions: (a) 600 second (s) incubation of anti-His probe in 2 μg/mL peptide or multiepitope antigen, (b) 1,800 s incubation in 10 μL of plasma diluted 1:19 with Q-Buffer diluent (GatorBio), and (c) 2,000 s incubation in a solution of 10 μg/mL goat anti-human IgG (Jackson ImmunoResearch). Steps 1 and 2 were followed by a 360 s wash in Q-Buffer. Endpoint nm shift measurements were normalized by subtracting the nm shift value after antigen loading wash (step 1) to account for any minor variation in the amount of immobilized antigen.

Anti*–T*. *cruzi* IgG BLI was performed on 335 BD specimens (*n* = 250, seropositive; *n* = 85, seronegative) to evaluate antibody reactivity to the peptide antigen as well as seroreactivity to the *T*. *cruzi* multiepitope with and without the TS23 peptide. Region-of-origin data were available for all seropositive specimens (Mexico, *n* = 92; Central America, *n* = 86; South America, *n* = 72).

### Receiver operating characteristic curve analysis.

The performance of TcMulti and TcMulti+TS23 using BLI was evaluated by receiver operating characteristic curve analysis with a 70/30 train/test split with equal proportions of seropositive and seronegative samples in across sets. Within the training set, we conducted a 5-fold cross-validation to assess the robustness of each antigen construct by calculating fold-specific AUC values.

### Statistics.

Associations between number of individual antibody targets and heart disease stage or region of infection were tested using Kruskal-Wallis tests. Motif analysis was performed using MEME and FIMO (34–36). Associations between BLI reactivity, serologic status, and region were tested using Kruskal-Wallis tests; significant results were followed with a post hoc MWU test with a correction for multiple comparisons using the Bonferroni method. A *P* value less than 0.05 was considered significant. In all figures, box plot horizontal lines represent the quartiles of the data, while whiskers show the rest of the distribution.

### Study approval.

Institutional review board approval for research use of deidentified human biospecimens was given by the University of California, San Francisco. The BD study protocol was approved by the institutional review board at the ARC. The CBM study protocol was approved by the Institutional Review Boards of Universidad Catolica Boliviana (Santa Cruz, Bolivia) and included consent for future use of deidentified specimens (40). NYBC specimens consisted of deidentified plasma obtained from adults who donated blood to the New York Blood Center.

### Data availability.

The graphed data in this manuscript can be accessed via the Supporting Data Values file. All raw and processed data are available for download at Dryad (https://doi.org/10.5061/dryad.9kd51c5v0). PhIP-seq analytical code is available at https://github.com/hkortbawi/tcruzi_phipseq_2025; commit ID 84d8009.

## Author contributions

JLD, JDW, CB, and JVR conceived the study. JDW, JLD, CB, JVR, HMK, RJM, EDG, NLB, JEP, WW, RLT, SLS, EEO, JES, EHC, RHG, and RPC developed methodology. HMK, RJM, JDW, JVR, A Mitchell, RAL, NLB, GW, A Maheshwari, AS, CJF, EAK, and ET investigated. HMK, RJM, and JVR performed formal analysis. HMK, RJM, JDW, and JVR visualized data. JLD and JDW acquired funding. JDW and JLD administered the project. JDW, JLD, CB, and EDG supervised. JDW, HMK, and RJM wrote the original draft. All coauthors reviewed and edited the manuscript.

This manuscript has 2 co–first authors. HMK is listed first for creating Figures 1–7; writing analytical code for Figures 2–7, Supplemental Figures 1–4, and Supplemental Figure 6; and performing the experiments that generated Figure 3. RJM is listed second for contributing analysis code for Supplemental Figures 5 and 7 and performing the experiments that generated Figures 5 and 6 and Supplemental Figure 8.

## Funding support

This work is the result of NIH funding, in whole or in part, and is subject to the NIH Public Access Policy. Through acceptance of this federal funding, the NIH has been given a right to make the work publicly available in PubMed Central.

- Chan Zuckerberg Biohub (JLD).
- Chan Zuckerberg Biohub Physician-Scientist Fellowship Program (JDW).
- NIH National Heart Lung and Blood Institute award K38HL154203 (JDW).
- NIH Eunice Kennedy Shriver National Institute of Child Health and Human Development award F30HD117526 (HMK).

## Supplementary Material

Supplemental data

ICMJE disclosure forms

Supporting data values

## Version 1 02/05/2026

In-Press Preview

## Version 2 03/16/2026

Electronic publication
