# Supplementary material for: A Trypanosoma cruzi trans-sialidase peptide demonstrates high serological prevalence among infected populations across endemic regions
Source: J Clin Invest. 2026 Feb 5;136(6):e199277. doi: 10.1172/JCI199277 (PMC12987611; doi:10.1172/JCI199277)
Supplement: Supplemental data [file jci-136-199277-s166.pdf]

SUPPLEMENTARY MATERIALS

Title: A *Trypanosoma cruzi* Trans-Sialidase Peptide Demonstrates High Serological Prevalence Among Infected Populations Across Endemic Regions

Table of Contents:

Figure S1: Histogram of read counts of the *T. cruzi* proteome PhIP-seq library

Figure S2: Glial fibrillary acidic protein (GFAP) enrichment is specific to polycolonal antibody control samples

Figure S3: Empirical determination of optimal threshold for calling peptides as seroreactive

Figure S4: Overlap in seroreactive peptides in blood donor (BD) and cardiac biomarker (CBM) specimen sets

Figure S5: Mass univariate analysis of PhIP-seq results

Figure S6: Antigen motifs

Figure S7. Receiver operating characteristic curve of TS23, TcMulti, and TcMulti-TS23 antigens.

Figure S8: Empirical determination of antigen concentration for optimal biolayer interferometry (BLI) performance

Table S1: Ranked list of *T. cruzi* PhIP-seq antigenic peptides by prevalence of enrichment in BD specimens

Table S2: Comparisons of regional seroreactivity to *T. cruzi* antigens by BLI

Table S3: Comparisons of seroreactivity to *T. cruzi* antigens by BLI

Table S4: Test performance characteristic comparison of novel multi-epitope antigen with and without TS23 by BLI immunoassay.

Authors: Hannah M. Kortbawi[1,2]†, Ryan J. Marczak[3,4]†, Jayant V. Rajan[1], Nash L. Bulaong[5], John E. Pak[5], Wesley Wu[5], Grace Wang[5], Anthea Mitchell[5], Aditi Saxena[5], Aditi Maheswari[3,4|], Rachel Alfaro Leone[4], Charles J. Fleischmann[4], Emily A. Kelly[4], Evan Teal[4], Rebecca L. Townsend[6], Susan L. Stramer[6], Emi E. Okamoto[7], Jacqueline E. Sherbuk[7], Eva H. Clark[8,9], Robert H. Gilman[10], Rony Colanzi[11], Efstathios D. Gennatas[3,12], Caryn Bern[3‡], Joseph L. DeRisi[1,5‡], Jeffrey D. Whitman[4‡*]

Affiliations:
[1]Department of Biochemistry and Biophysics, University of California San Francisco; San Francisco, CA, USA.
[2]Medical Scientist Training Program, University of California San Francisco; San Francisco, CA, USA.
[3]Department of Epidemiology and Biostatistics, University of California San Francisco; San Francisco, CA, USA.

[4]Department of Laboratory Medicine, University of California, San Francisco; San Francisco, CA, USA.
[5]Chan Zuckerberg Biohub San Francisco; San Francisco, CA, USA.
[6]Scientific Affairs, American Red Cross; Gaithersburg, MD, USA.
[7]New York University School of Medicine; New York, NY, USA.
[8]Section of Infectious Diseases, Department of Medicine, Baylor College of Medicine; Houston, TX, USA.
[9]Division of Tropical Medicine, Department of Pediatrics, Baylor College of Medicine; Houston, TX, USA.
[10]Johns Hopkins Bloomberg School of Public Health; Baltimore, MD, USA.
[11]Universidad Catolica Boliviana; Santa Cruz, Plurinational State of Bolivia.
[12]Department of Medicine, University of California San Francisco; San Francisco, CA, USA.
†, Authors contributed equally.
‡, Shared senior authorship
*, Corresponding author, jeffrey.whitman@ucsf.edu, Phone: 415-476-1000, Address: 2540 23rd St, San Francisco, CA 94110

JVR current affiliation, Current affiliation Pfizer, Inc; Collegeville, PA, USA.
AS current affiliation, Department of Immunology and Infectious Diseases, Harvard T. H. Chan School of Public Health; Boston, MA, USA.
AM current affiliation, Keck School of Medicine, University of Southern California, Los Angeles, CA, USA.
CJF current affiliation, Touro University, Vallejo, CA, USA.
EEO current affiliation, Independent Consultant.
JES current affiliation, University of South Florida; Tampa, FL, USA.
SLS current affiliation, Infectious Disease Consultant, North Potomac, MD

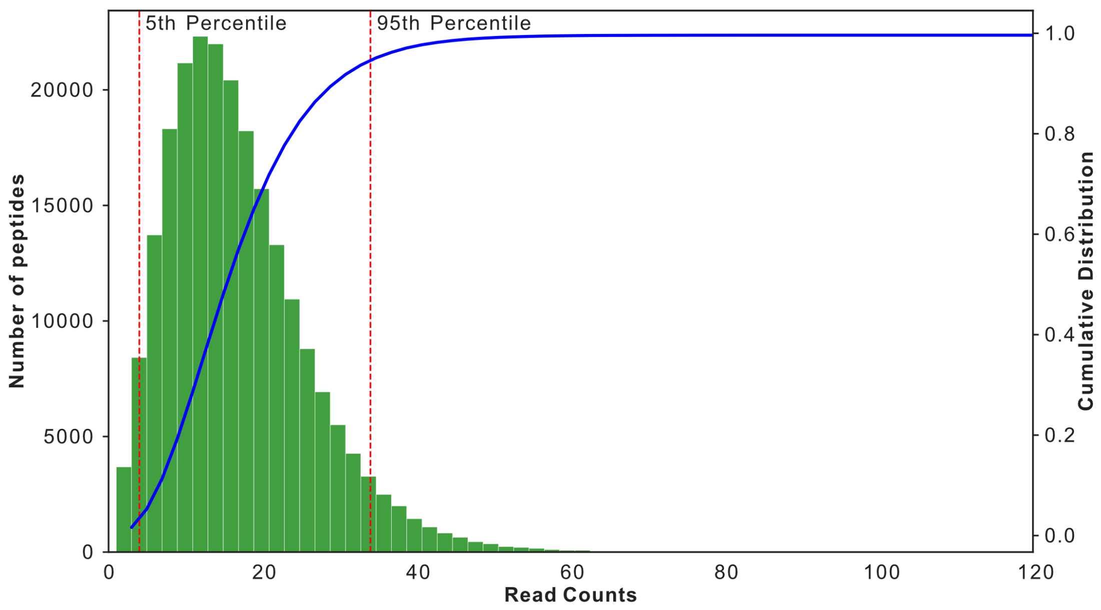

Figure S1. Histogram of read counts of the *T. cruzi* proteome PhIP-seq library. Read counts corresponding to the 5th and 95th percentile in the distribution (marked by red dotted lines) are within 9-fold of each other. Cumulative density plot of the distribution is shown in blue.

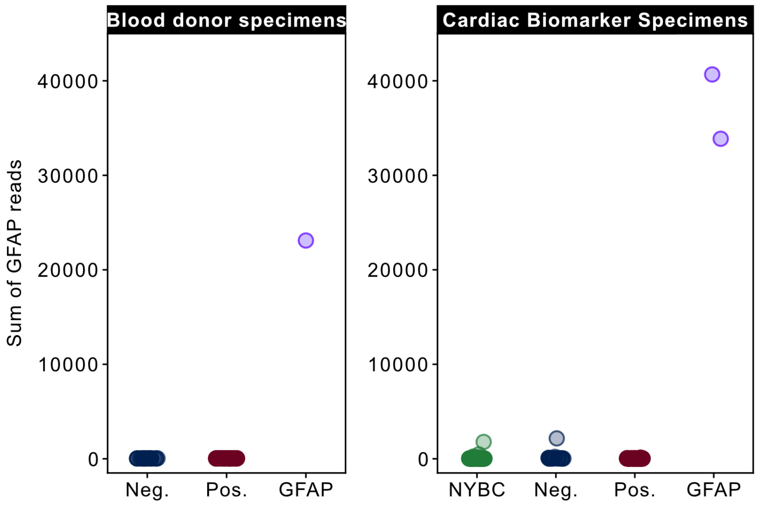

Figure S2. Glial fibrillary acidic protein (GFAP) enrichment is specific to polyclonal antibody control samples. GFAP enrichment is plotted as the sum of read counts for all peptides in the *T. cruzi* PhIP-seq library that are part of the GFAP protein. In both PhIP-seq runs, only sample wells with polyclonal antibody to GFAP showed increased GFAP reads.

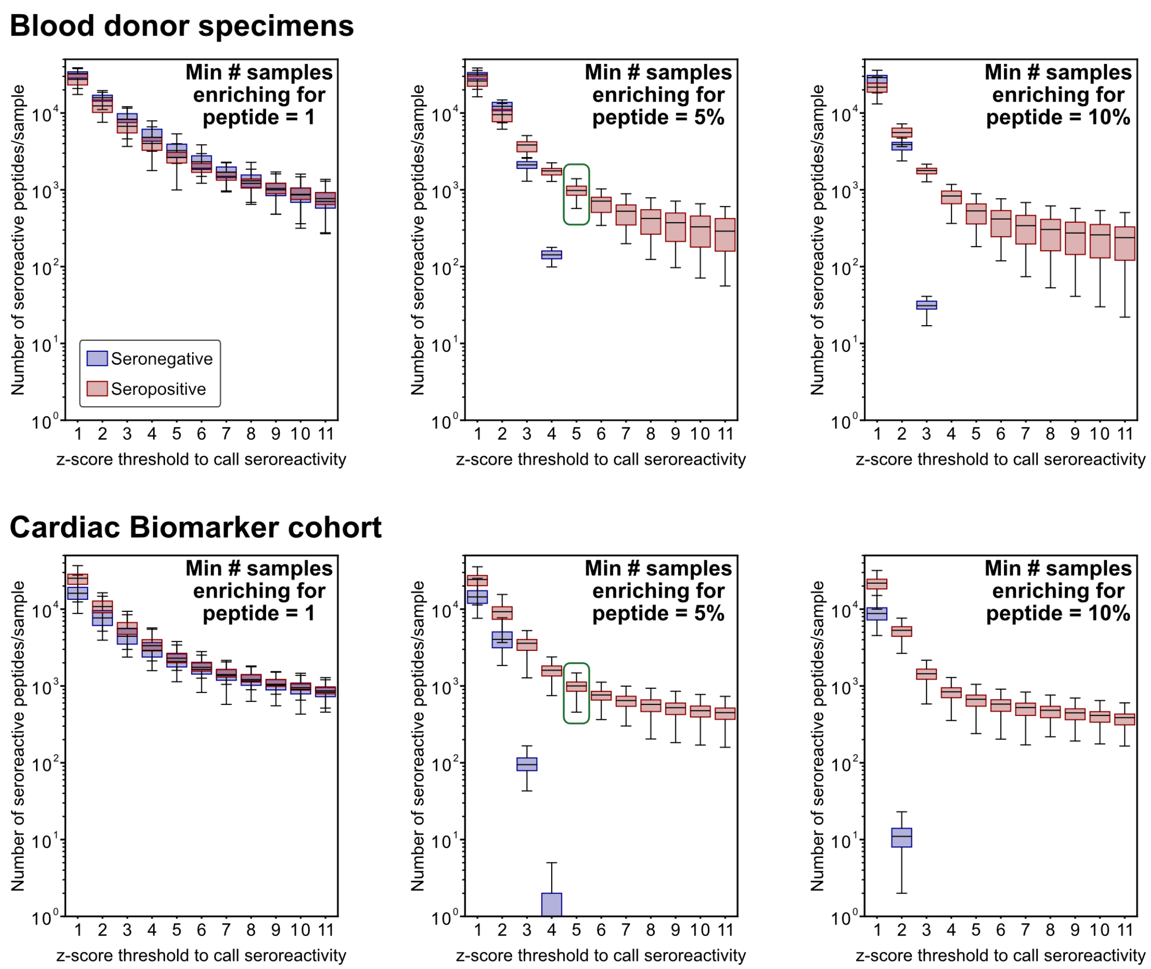

Figure S3. Empirical determination of optimal threshold for calling peptides as seroreactive. Box plots showing the number of seroreactive peptides per specimen at each threshold of antigen sharing (n=1, 5%, or 10% of seropositive specimens) and at z-score cutoffs of 1-11 over the mean in seronegative specimens. The final threshold for calling seroreactivity is indicated by the green boxes and was selected to minimize the number of seroreactive peptides in seronegative samples.

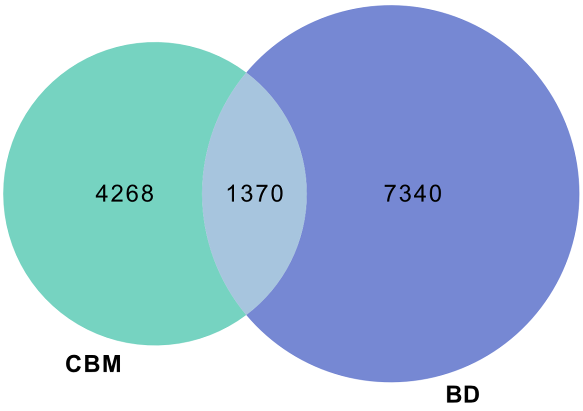

Figure S4. Overlap in seroreactive peptides in blood donor (BD) and cardiac biomarker (CBM) specimen sets.

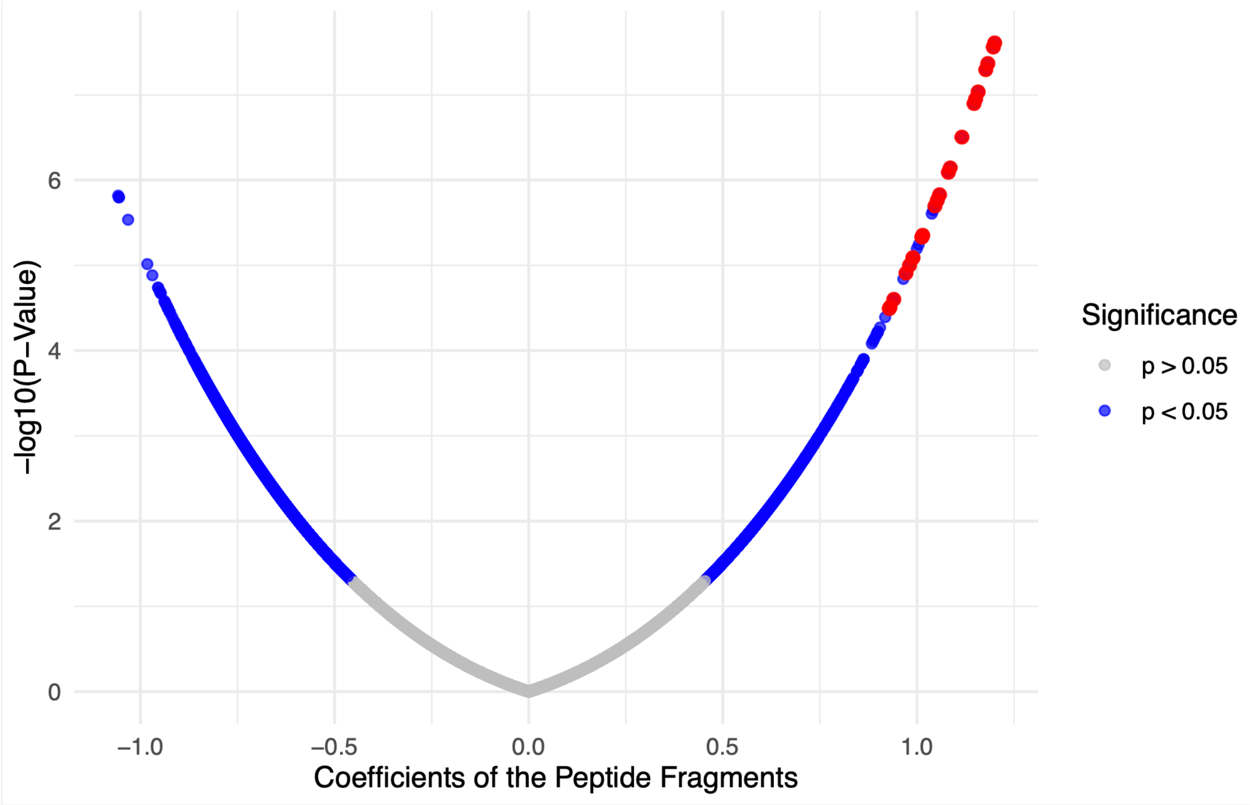

Figure S5. Mass univariate analysis of PhIP-seq results. This volcano plot shows the results of mass univariate analysis. The coefficient refers to the term given by the linear regression result. Coefficients>0 are associated with positive specimens. Each dot represents a single peptide, and blue dots have a *p*-value < 0.05. Red dots represent peptides that were identified as seroreactive in ≥90% of BD specimens by z-score analysis and $p < 0.05$ by mass-univariate analysis (n=23 peptides). A ranked list of peptides is available in Table S1.

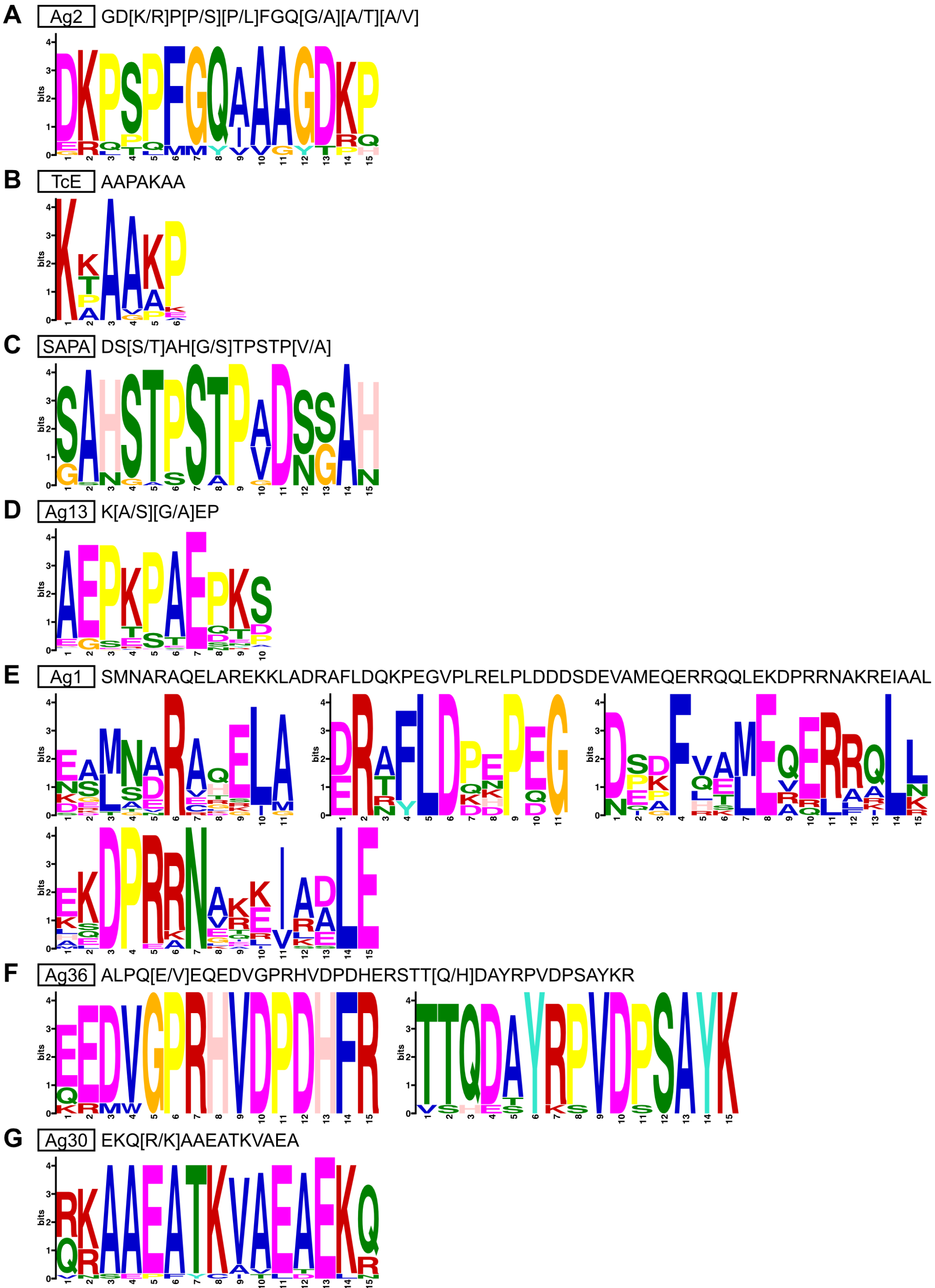

Figure S6. Antigen motifs. Multiple EM for Motif Elicitation (MEME) analysis was performed on seroreactive peptides that mapped to a known antigen protein (e.g., all nucleoporin peptides were analyzed to find the Ag2 motif). The output motifs are shown here, with the reference sequence (*24*) for the given antigen in plain text above the motif diagram.

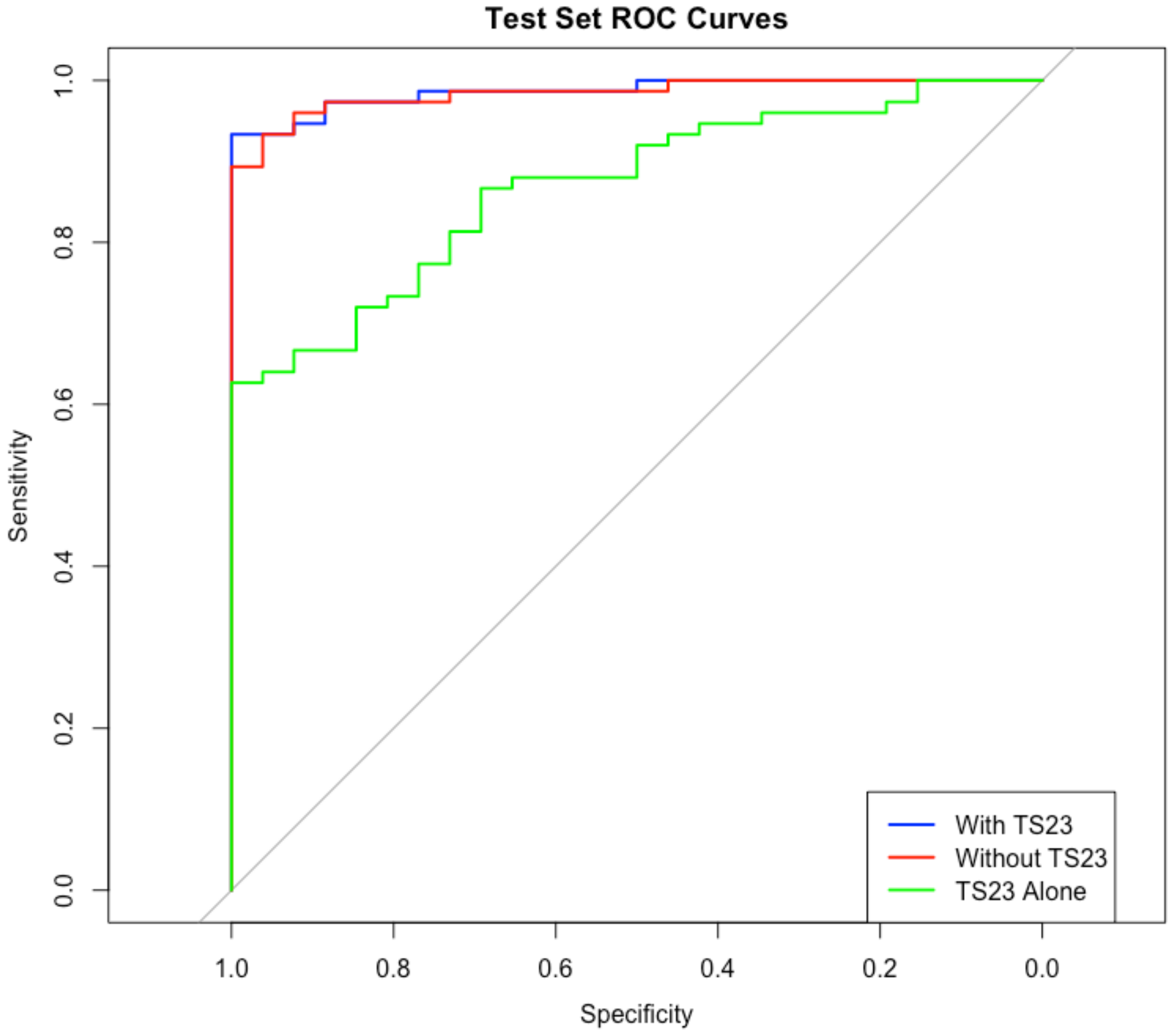

Figure S7. Receiver operating characteristic curve of TS23, TcMulti, and TcMulti-TS23 antigens. The performance of TcMulti, TcMulti-TS23, and TS23 using BLI were evaluated by ROC curve analysis. The dataset was first split into training (70%) and held-out test (30%) sets with equal proportions of seropositive and seronegative samples. Antigen robustness was assessed using 5-fold cross-validation within the training set by calculating fold-specific AUC values. Final performance was evaluated on the held-out test set using ROC curve analysis, and the ROC curve shown corresponds to performance on the test-set. The test-set AUCs were as follows: TS23, 0.869; TcMulti,0.984; TcMulti-TS23, 0.986.

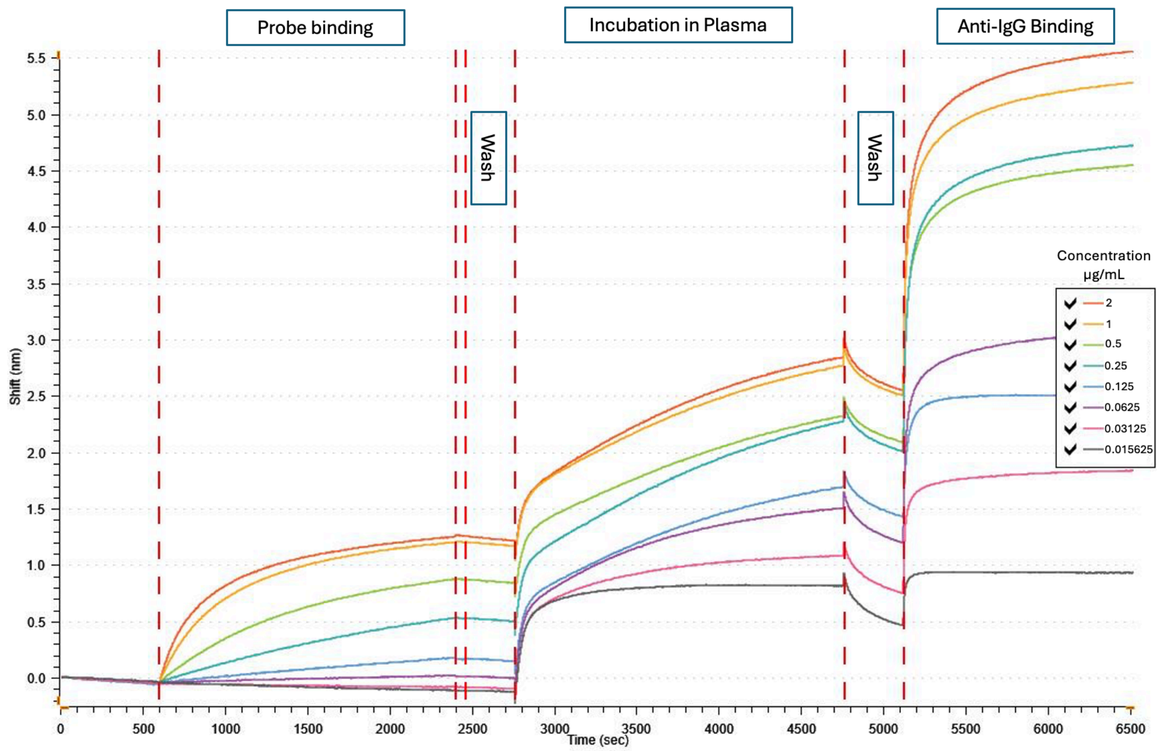

Figure S8. Empirical determination of TS23 antigen concentration for optimal biolayer interferometry (BLI) performance. Eight protein concentrations were tested, ranging from 0.015625 µg/mL to 2 µg/mL. 2 µg/mL was selected as the optimal concentration for further assays.

Table S1. Ranked list of *T. cruzi* PhIP-seq antigenic peptides by prevalence of enrichment in blood donor specimens.

Protein Name	% Seropositive (n)	RefSeq ID	Sequence
Trans-sialidase, putative	100% (64)	XP_804569.1	GGADAAPTPSSAAPGETKVPSELNATIPSDHDILLEFRDLAAMALIG
Trans-sialidase, putative	98% (63)	XP_817098.1	TAPGETKIPSELNATIPSDHDILLEFRELAAMALIGDSTVHVCVSRV
Surface antigen 2 (CA-2), putative	97% (62)	XP_813516.1	PFGQAAAGDKPSPFGQAAAGDKPSPFGQAAAGDKPSPFGQAAAGDKP
Surface antigen 2 (CA-2), putative	97% (62)	XP_818927.1	AAAGDKPPPFGQAAAGDKPAPFGQAAAGDKLSLFGQAAAGDKPSPFG
Surface antigen 2 (CA-2), putative	97% (62)	XP_813516.1	AAAGDKPSPFGQAAAGDKQPPFGQAAAGDKKPPFGQAAAGDKPSPFG
Surface antigen 2 (CA-2), putative	97% (62)	XP_813516.1	AAAGDKPPPFGQAAADDKPSPFGQAAAGDKPPPFGQAAAGDKPPPFG
Surface antigen 2 (CA-2), putative	97% (62)	XP_813516.1	DKPPPFGQAAAGDKPPPFGQAAAGDKPSPFGQAAAGDKPSPFGQAAA
Surface antigen 2 (CA-2), putative	97% (62)	XP_813516.1	DKPSPFGQIAAGDKPSPFGQVAAGDKPSPFGQAAAGDKPPPFGQAAA
Surface antigen 2 (CA-2), putative	95% (61)	XP_813516.1	TLEKTQTEQKTEPFGQAAADDKPSPFGQAAAGDKPPPFGQAAADDKP
Surface antigen 2 (CA-2), putative	95% (61)	XP_813516.1	PFGQAAADDKPSPFGQAAAGDKPSPFGQIAAGDRPPPFGQAAAGDKQ
Surface antigen 2 (CA-2), putative	95% (61)	XP_818927.1	GKPSPFGQAAAGDKPSPFGQGTAFDASRSTVFANAPGVAQVSFGKPS
Surface antigen 2 (CA-2), putative	95% (61)	XP_818927.1	DKLSLFGQAAAGDKPSPFGQAAAGDKPSLFGQAAAGDKPSPFGQAAA
60S ribosomal protein L23a, putative	95% (61)	XP_820993.1	MPAKTAVSKAAAPQKAAAPKKAAAPQKAAAPKKAAAQQKAAVAKKAV
Surface antigen 2 (CA-2), putative	95% (61)	XP_813516.1	PFGQAAAGDKQPPFGQAAAGDKQPPFGQAAAGDKPSPFGQIAAGDRP
Surface antigen 2 (CA-2), putative	95% (61)	XP_813516.1	PFGQAAAGDKPSPFGQVAAGDKPSPFGQVAAGDKPSLFGQGTGFDAS
Surface antigen 2 (CA-2), putative	94% (60)	XP_813516.1	IAAGDKPSPFGQVAAGDKPSLFGQAAAGDKPSPFGQVAAGDKPSLFG
Surface antigen 2 (CA-2), putative	94% (60)	XP_813516.1	AAAGDKPSPFGQAAAGDKPSPFGQAAAGDKPSPFGQIAAGDKPSPFG
Surface antigen 2 (CA-2), putative	94% (60)	XP_813516.1	DKQPPFGQAAAGDKQPPFGQAAAGDKQPPFGQAAADDKPSPFGQAAA
Surface antigen 2 (CA-2), putative	92% (59)	XP_813516.1	AAAGDKPSPFGQIAAGDKPSPFGQAAAGDKQPPFGQAAAGDKQPPFG
Surface antigen 2 (CA-2), putative	92% (59)	XP_813516.1	LFVPIAAGDKPSPFGQIAAGDKPSLFGQAAAGDKPSPFGQVAAGDKQ
Surface antigen 2 (CA-2), putative	91% (58)	XP_813516.1	IAAGDRPPPFGQAAAGDKQPPFGQAAAGDKQPPFGQAAAGDKQPPFG
Surface antigen 2 (CA-2), putative	91% (58)	XP_813516.1	AAAGDKPSPFGQIAAGDRPPLFVPIAAGDKQPPFGQAAAGDKQPPFG
Surface antigen 2 (CA-2), putative	91% (58)	XP_813516.1	DKQPPFGQAAAGDKQPPFGQIAAGDRPPLFVPIAAGDKPSPFGQIAA

Table S2. PhIP-seq antibody reactivity of current diagnostic antigens and TS23 in Chagas disease seropositive specimens. Percent of samples enriched (z-score ≥5) for recombinant antigens in current FDA-cleared serology tests in blood donor (BD) and cardiac biomarker (CBM) specimen sets. Each antigen motif was derived using Multiple EM for Motif Elicitation (MEME) and then scored against the entire T. cruzi PhIP-seq proteome. The maximum z-score across all peptides with significant sequence matches to a given antigen motif was used to define positive reactivity (z-score ≥5) for each sample and each antigen.

**Antigen**	**BD Prevalence** (*n* = 64)	**CBM Prevalence** (*n* = 114)
Ag2	98%	97%
TcE	98%	95%
Ag1	95%	98%
SAPA	95%	93%
Ag13	94%	82%
Ag36	89%	96%
Ag30	47%	77%
KMP-11	6%	12%
TS23	100%	95%

Table S3. Comparisons of regional seroreactivity to *T. cruzi* antigens by BLI. A Kruskal-Wallis test was used to compare seroreactivity to each *T. cruzi* antigen across regions of infection. There was a statistically significant difference in reactivity between regions for each antigen: TS23 $H(3)=154.760$, $p<0.0001$; TcMulti $H(3)= 201.428$, $p<0.0001$; TcMulti-TS $H(3)= 206.263$, $p<0.0001$. Post-hoc Mann-Whitney U-tests with Bonferroni were used to compare regional seroreactivity to each antigen. *P*-values were adjusted for multiple comparisons using the Bonferroni correction; adjusted *p*-values are reported below; *p*-values <0.05 are in bold. Mexico, n=92; Central America, n=86; South America, n=72; Negative, n=85.

	TS23				TcMulti				TcMulti-TS23			
	Mexico	C. America	S. America	Negative	Mexico	C. America	S. America	Negative	Mexico	C. America	S. America	Negative
Mexico		**9.44E-05** (2472)	**2.14E-04** (2064)	**4.60E-17** (6841)		**4.29E-03** (2793)	**3.79E-10** (1339)	**3.01E-26** (7577)		**1.17E-03** (2672)	**2.78E-10** (1325)	**3.60E-27** (7643)
C. America	**9.44E-05** (2472)		1 (NS) (3233)	**2.05E-23** (370)	**4.29E-03** (2793)		**1.09E-03** (2023)	**3.03E-27** (102)	**1.17E-03** (2672)		**2.26E-03** (2077)	**4.71E-28** (48)
S. America	**2.14E-04** (2064)	1 (NS) (3233)		**2.75E-22** (252)	**3.79E-10** (1339)	**1.09E-03** (2023)		**3.86E-25** (71)	**2.78E-10** (1325)	**2.26E-03** (2077)		**2.65E-25** (61)
Negative	**4.60E-17** (6841)	**2.05E-23** (370)	**2.75E-22** (252)		**3.01E-26** (7577)	**3.03E-27** (102)	**3.86E-25** (71)		**3.60E-27** (7643)	**4.71E-28** (48)	**2.65E-25** (61)	

Table S4. Comparisons of seroreactivity to *T. cruzi* antigens by BLI. A Kruskal-Wallis test was used to compare seroreactivity to each *T. cruzi* antigen among a region of infection. There was a statistically significant difference in reactivity to *T. cruzi* antigens for each region: Mexico $H(2)= 7.821$, $p=0.02$; Central America $H(2)=7.263$, $p=0.027$; South America $H(2)= 45.171$, $p<0.0001$; Seronegative $H(2)= 124.329$, $p<0.0001$. Post-hoc Mann-Whitney U-tests with Bonferroni were used to compare regional seroreactivity to each antigen. *P*-values were adjusted for multiple comparisons using the Bonferroni correction; adjusted *p*-values are reported below with U values in parentheses. Mexico, n=92; Central America, n=86; South America, n=72; Negative, n=85.

	Mexico			Central America			South America			Negative		
	TS23	TcMulti	TcMulti-TS23	TS23	TcMulti	TcMulti-TS23	TS23	TcMulti	TcMulti-TS23	TS23	TcMulti	TcMulti-TS23
TS23		0.06 (NS) (3387)	**0.04** (3331)		0.15 (NS) (3055)	**0.03** (2855)		**3.86E-08** (1168)	**1.02E-08** (1112)		**3.03E-19** (6529)	**3.03E-22** (6761)
TcMulti	0.06 (NS) (3387)		1 (NS) (4185)	0.15 (NS) (3055)		1 (NS) (3510)	**3.86E-08** (1168)		1 (NS) (2456)	**3.03E-19** (6529)		**8.40E-03** (4572)
TcMulti-TS23	**0.04** (3331)	1 (NS) (4185)		**0.03** (2855)	1 (NS) (3510)		**1.02E-08** (1112)	1 (NS) (2456)		**3.03E-22** (6761)	**8.40E-03** (4572)	

Table S5. Test performance characteristic comparison of novel multi-epitope antigen with and without TS23 by BLI immunoassay. A fixed-cutoff approach to determine qualitative test performance (sensitivity and specificity to original blood donor testing result) was calculated using 2 standard deviations above the average reactivity of seronegative specimens to TcMulti-TS23 and TcMulti as the threshold for positive seroreactivity, yielding a nm shift cutoff 0.27.

	**Sensitivity (95% CI)[a]**	**Specificity (95% CI)[a]**
**TcMulti-TS23[b]**	85.6% (80.7%, 89.4%)	98.8% (93.6%, 99.9%)
**TcMulti[b]**	85.2% (80.3%, 89.1%)	97.7% (91.8%, 99.6%)

[a] 95% Confidence Intervals (CI) calculated using binomial exact.

[b] TcMulti is a multi-epitope construct based on consensus sequences of classical T. cruzi serology antigens. TcMulti-TS23 is the consensus classical multi-epitope with TS23.
